# Functional material probes and advanced technologies in organ-on-a-chip characterization

**DOI:** 10.7150/thno.122552

**Published:** 2026-01-01

**Authors:** Yanan Wang, Xiao Chang, Shiwen Deng, Shihuan Tang, Peng Chen

**Affiliations:** 1 Experimental Research Center, China Academy of Chinese Medical Sciences, Beijing 100700, China.; 2 Institute of Chinese Materia Medica, China Academy of Chinese Medical Sciences, Beijing 100700, China.; 3 Department of Urology, Chinese People's Liberation Army (PLA) General Hospital/PLA Medical School, Beijing 100039, China.; 4 State Key Laboratory for Quality Ensurance and Sustainable Use of Dao-di Herbs, Beijing 100700, China.; 5 Analysis of Complex Effects of Proprietary Chinese Medicine, Hunan Provincial Key Laboratory, Yongzhou, Hunan Province 425199, China.

**Keywords:** functional material probes, organ-on-a-chip, biosensors, characterization techniques

## Abstract

Organ-on-a-Chip (OoC) integrates advanced biomaterials, microfluidics, and cell biology to simulate organ structures and dynamic microenvironments, offering a vital platform for drug development and disease modeling. The miniature scale, structural precision, high biomimetic fidelity, and inherent dynamic complexity of OoCs pose challenges for conventional offline detection methods, including molecular-specific detection and omics analysis. Online detection technologies, particularly fluorescence probes and biosensors, address this limitation by enabling *in situ*, non-invasive, and real-time monitoring with high spatiotemporal resolution. As the core platform for microphysiological systems, the accuracy with which OoCs replicate human organ function and its capability for dynamic characterization are depend on breakthroughs in the performance of current probe and sensing materials. Now, comprehensive reviews specifically dedicated to fluorescence probe techniques and its application in real-time characterization of OoCs remain lacking. Herein, to address this gap, we establish an offline-online detection technologies multidimensional evaluation framework in OoCs characterization. Furthermore, we integrate the burgeoning application of image recognition technologies and artificial intelligence (AI) within the OoCs. Our aim is to provide methodological support for advancing OoCs standardization and industrial quality control, and to stimulate the development of OoCs into more reliable and effective tools for biomedical research and applications. The ongoing maturation and industrialization of OoC technology will solidify its role as a transformative tool, bridging foundational research and clinical applications with enhanced reliability and efficacy.

## 1. Introduction

Organ-on-a-Chip (OoC) technology represents a revolutionary *in vitro* microphysiological systems (MPSs). Its core concept involves using microfluidic technology to construct 3D tissue structures incorporating human cells or organoids on a microfluidic chip, thereby mimicking key functions of human organs 1. Unlike basic microfluidic chips that simply handle fluid flow, OoCs employ biomimetic designs, such as microchannels and functional membranes, to enable dynamic fluid perfusion. This allows them to simulate physiological microenvironments, including blood shear stress and mechanical tissue forces, while supporting co-culture of multiple cell types to reproduce organ-level interactions 2. This approach fundamentally overcomes the limitations of traditional models. Compared to two-dimensional (2D) static cell cultures, which lack there-dimensional (3D) structure and physiological relevance, OoCs provide more biologically meaningful readouts of cell behavior and tissue function 3. In contrast to animal models, which exhibit significant species-specific differences, OoCs utilize human cells, including those derived from primary or stem cell sources. They not only substantially reduce reliance on animal experiments but also align with the 3R (replacement, reduction, and refinement) principle of animal research ethics, while enabling more direct prediction of human-specific physiological and pathological responses. This capability is critical for addressing the high failure rate of drug candidates in human trials, where over 90% of preclinically successful compounds fail clinically 4. Thus, engineering 3D human tissue models is essential for accurate assessment of drug metabolism, toxicity, and efficacy.

The high predictive power of OoCs underscores their translational value. Key applications include: precise assessment of organ-specific toxicity (e.g., cardiotoxicity, hepatotoxicity) in toxicology and safety pharmacology; functional integration into drug discovery pipelines to improve compound screening efficiency; the creation of multi-OoCs systems, also called “human-on-a-chip”, for studying systemic drug absorption, distribution, metabolism, excretion, and pharmacokinetics (ADME/PK), as well as biological barrier function; and disease modeling and therapeutic efficacy evaluation by reconstituting pathological conditions and testing personalized treatment strategies 5. In 2025, U.S. regulatory and research agencies introduced landmark policies to advance more human-relevant alternative methods. In April, the Food and Drug Administration (FDA) announced plans to gradually phase out animal testing requirements for monoclonal antibodies and other drugs 6. Subsequently, in July, the National Institutes of Health (NIH) stated that it would no longer fund new research projects relying solely on animal experiments 7. This strategic shift creates a pivotal opportunity for advanced technologies such as OoCs. By enabling rapid toxicity screening and integrating AI for accurate drug response prediction, OoC technology is emerging as a key tool to bridge the translational gap in drug development.

The microenvironment within OoC systems is inherently complex and dynamic. These systems must not only replicate the structures of multiple cell types and tissues but also simulate their interactions and behaviors under physiological conditions 8. Crucially, these interactions are highly time-dependent, requiring models where states evolve over time. Such dynamic complexity renders traditional offline detection methods, reliant on fixed-time-point sampling, insufficient for validation, thereby imposing an urgent need for real-time, continuous monitoring technologies. Furthermore, OoCs development faces technical bottlenecks, particularly in multi-organ functional integration and processing the massive, multi-dimensional data generated by high-precision, real-time sensing 9. Addressing these challenges demands interdisciplinary collaboration to achieve key breakthroughs in high-precision characterization technologies for biomarker detection, advanced system integration, and complex data analysis 10. These advances are essential to ensure the model's biological functions align with human organ physiology. In traditional characterization methods for OoCs, the chip output can be connected to analytical devices such as high-performance liquid chromatography (HPLC) or mass spectrometers (MS), or rely on biochemical detection means like DNA assays or reagent kits to achieve precise identification and quantitative analysis of biomarkers. While these traditional molecular-specific biomarker detection and omics analysis offer high sensitivity and molecular mechanism resolution, they exhibit significant material limitations: destructive sampling requiring fixation or lysis of OoCs, which precludes real-time monitoring of cellular dynamic behaviors; invasive probes or extraction interfaces that disrupt the homeostasis of physiological microenvironments; and operational complexities such as high sample consumption and increased labor costs due to multi-step processing 11.

The accuracy of OoCs in simulating human organ functions and its dynamic characterization capabilities fundamentally depend on breakthroughs in the performance of probe and sensing materials. Among various functional materials, fluorescent probes have become a key technology for realizing real-time tracking of molecular dynamics in microscale microenvironments, owing to their specific fluorescent labeling capabilities for intracellular ions, metabolites, and biomarkers. Such probes can capture transient biochemical signals in the microenvironment with nanoscale spatial resolution, such as monitoring the contraction activities of cardiac myocytes using calcium-sensitive fluorescent probes or tracking the dynamic activation of signaling pathways through FRET probes 12. Integrated microfluidic sensor devices, leverage piezoelectric materials, nanoelectrodes, flexible bioelectronic materials, and optical sensing components to measure various physiological and environmental parameters within OoCs 13. Compared with the limitations of traditional offline detection methods that require sample destruction, online detection technologies represented by fluorescent probes enable continuous monitoring of dynamic processes through non-invasive labeling. Notwithstanding their critical role in OoCs functionalization, sensors and fluorescent probes face limitations in application: physical sensors suffer from spatial constraints and vulnerability to biological contamination during miniaturized integration, and their difficulty in *in situ* calibration leads to poor long-term stability; fluorescent probes are deeply troubled by background autofluorescence interference and photobleaching effects, while their potential cytotoxicity and the uncertainty of concentration distribution in dynamic microenvironments also restrict quantitative reliability. Furthermore, both physical sensors and fluorescent probes may perturb the delicate microenvironment simulated by OoCs due to their invasiveness, and achieving high-sensitivity, wide dynamic range, and multi-parameter synchronous detection remains technically challenging. These factors collectively result in difficulties in data standardization and cross-platform comparability for OoCs detection, constituting bottlenecks for the technology's in-depth development and broad application. Thus, an ideal characterization strategy must integrate the advantages of online and offline technologies. This requires synergistically utilizing traditional offline methods to achieve comprehensive and in-depth biological mechanism analysis, while combining the continuous dynamic process information provided by real-time online technologies such as sensors and probes. Through data fusion and cross-validation, it is possible to achieve a truly comprehensive evaluation and optimization of OoCs.

Research on OoC platforms has witnessed steady growth in recent years, reflected in numerous reviews. While many of these focus on manufacturing materials and integration of OoCs 14-17, and others address the application of biosensors for OoCs optimization and validation 18, 19, comprehensive reviews specifically dedicated to fluorescence probe techniques and their application in real-time characterization of OoCs remain conspicuously lacking. To address this gap, this paper establishes a focused discussion framework centered on fluorescence probes and biosensors. Firstly, we provide a comparative analysis of real-time online detection technologies alongside traditional offline detection methods employed in OoCs characterization. Furthermore, we integrate the burgeoning application of image recognition technologies and AI within the OoCs. Finally, by investigating the principles and application scenarios of these mainstream characterization techniques, our aim is to provide methodological support for advancing OoCs standardization and industrial quality control, and to stimulate the development of OoCs into more reliable and effective tools for biomedical research and applications.

## 2. Online Detection Technologies in OoCs

Online detection technology, leveraging its real-time, dynamic, and continuous monitoring capabilities, has emerged as a pivotal tool for addressing the challenges of precisely characterizing and validating dynamic physiological processes within OoCs. Compared to traditional offline methods reliant on destructive sampling, its core advantages lie in its* in situ*, non-invasive nature and high spatiotemporal resolution, enabling the non-invasive capture of transient physiological activities of cells/tissues, the dynamic evolution of complex signaling networks, and the spatiotemporal response processes to drugs, thereby enhancing the biomimetic fidelity and reliability of OoCs. From the perspectives of technical principles and application dimensions, the core components of current online detection systems primarily include fluorescent probe technology utilizing functional dyes or nanoscale probe materials to achieve highly specific labeling and dynamic imaging of molecular targets; biosensors employing advanced transducer elements such as nanoelectrodes, piezoelectric materials, and flexible bioelectronics to convert biological signals into quantifiable outputs; image recognition systems combining computer vision algorithms for the automated analysis of cell morphology and behavior; and AI-driven analytics platforms integrating multi-dimensional data streams and leveraging AI algorithms for in-depth interpretation. Despite immense potential, existing technologies still face challenges in areas such as miniaturization and integration, long-term stability, resistance to environmental interference and the synchronous, precise detection of multiple parameters. The key to overcoming these bottlenecks and achieving a performance leap fundamentally lies in the innovative design and engineering application of novel functional materials, including probe materials with superior photostability, interface materials with high biocompatibility/anti-fouling properties, and nanocomposite materials integrating multi-sensing functions. These material-driven technological advancements are collectively building an increasingly sophisticated online detection framework, laying a solid technical foundation for the deeper development of OoCs research.

### 2.1 Biosensors in OoCs

Breakthroughs in functional materials have established diverse biosensors, including optical, electrochemical, and mechanical types, as core tools for achieving* in situ*, real-time monitoring of critical biomarkers and microenvironmental factors within OoCs. Optical biosensors rely on specific optical materials such as plasmonic metal nanostructures, fluorescent/Raman labels, photonic crystals, and fiber-optic coating materials. They achieve high sensitivity, strong selectivity, and real-time monitoring capabilities by precisely detecting light signal changes (e.g., intensity, wavelength, and refractive index) induced by interactions between light and biorecognition elements 20. Electrochemical sensors utilize functionalized electrode materials, including nanostructured carbon materials, metal/metal oxide nanoparticles, and conductive polymers, to convert target recognition events into electrical signals. Benefiting from the excellent electrocatalytic activity, large specific surface area, and modifiability of these materials, they exhibit high sensitivity, low detection limits, rapid response times, and operational simplicity, leading to widespread application 21. Electrophysiological sensors primarily employ microelectrode arrays (MEAs) or flexible/stretchable bioelectronic materials. These directly detect electrical activities (e.g., action potentials, field potentials) or impedance changes generated by cells or tissues, providing unique insights into neural activity, cardiac contraction, and intercellular electrical signaling crucial for understanding biological function and disease mechanisms 22. Mechanical sensors operate on physical principles such as piezoresistive, piezoelectric, or capacitive effects to convert specific mechanical stimuli, including the contractile force of cardiac tissues, fluid shear stress on vessel walls, and hydrostatic pressure within lumens, into measurable electrical signals. This capability provides researchers with continuous, quantitative data on dynamic tissue function, thereby overcoming the limitations of conventional endpoint analysis 23. In this discussion, we categorized electrophysiological sensors as a subset of electrochemical sensors. By integrating material platforms based on these distinct sensing principles, these sensors synergistically enable multi-dimensional, comprehensive monitoring of complex biological systems and environmental factors within OoCs. This provides powerful support for drug screening and disease model research.

#### 2.1.1 Optical sensors in OoCs

As a vital component of the online detection technology system, optical sensors based on advanced optical materials play an indispensable role in OoCs due to their inherent high sensitivity, non-contact measurement capability, and real-time dynamic monitoring. The core principle of these sensors lies in the interaction of light with matter, particularly functional optical materials. By precisely detecting changes in parameters such as light intensity, wavelength, phase, or polarization state, they enable quantitative analysis of target biomarkers or environmental factors 24.

**Figure 1:**
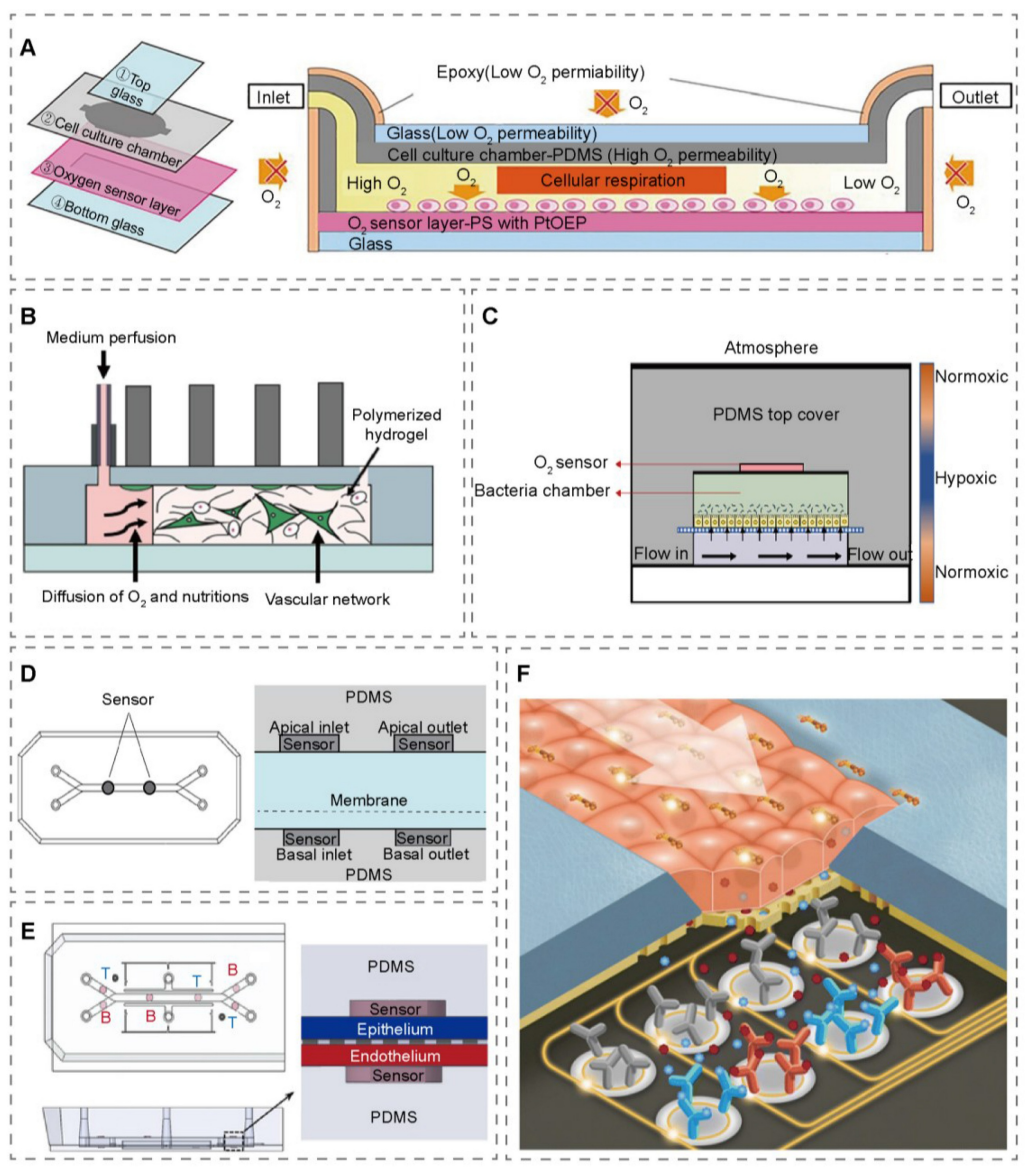
** Representative examples of optical sensors implementation in OoCs.** (A) A novel hepatic culture device with an integrated oxygen sensor. Adapted with permission from 27, copyright 2018, Elsevier. (B) A gas-impermeable 3D cell culture chip with integrated oxygen sensor. Adapted with permission from 28, copyright 2018, Frontiersin.org. (C) A two-chamber PDMS microfluidic device with integrated oxygen sensor. Adapted with permission from 30, copyright 2021, Wiley. (D) A schematic of the oxygen sensing Intestine Chip with four oxygen sensors. Adapted with permission from 31, copyright 2022, Royal Society of Chemistry. (E) An oxygen-sensitive human intestinal chip microfluidic culture device with 6 oxygen-quenched fluorescent particles embedded. Adapted with permission from 32, copyright 2019, Springer Nature. (F) A photonic sensor-enabled tissue chip. Adapted with permission from 34, copyright 2024, Royal Society of Chemistry.

Optical oxygen-sensing technology demonstrates unique advantages in simulating and monitoring the critical microenvironmental oxygen gradients within OoCs. Its performance heavily depends on the phosphorescent oxygen-sensitive materials employed 25. Currently, phosphorescent microparticles/films based on platinum porphyrin/platinum porphyrin derivatives (e.g., Pt (OEP), Pt-TPTBPF, and Pt-TFPP) represent the mainstream. These microparticles operate based on the principle of phosphorescence quenching: when oxygen molecules in the environment interact with the phosphorescent oxygen-sensitive microparticles, the paramagnetic properties of oxygen promote a non-radiative transition from the excited state to the ground state, leading to a decrease in phosphorescence intensity. Detecting this phosphorescence signal decay, emitted by the specific oxygen-sensitive material, allows for high-precision, rapid-response perception of oxygen concentration 26. Benefiting from the excellent photophysical properties and processability of these materials (e.g., embeddable in polymer matrices like polydimethylsiloxane (PDMS)), phosphorescent oxygen-sensitive material-based technology offers core advantages including non-invasiveness, rapid response, and the provision of real-time dynamic feedback. Satomi et al. embedded Pt (OEP) oxygen-sensitive material within a glass mask coated with PDMS at the bottom of a liver-on-a-chip, successfully replicating a physiological liver lobule oxygen gradient *in vitro* using fluorescence intensity modulation (Figure 1A) 27. Helene employed Pt-TPTBPF complexes as the sensing material to achieve time-resolved monitoring of oxygen partial pressure in 2D/3D OoCs. This allowed estimation of the oxygen consumption rate in cell monolayers under conditions of increased flow rate, higher cell numbers, varied cell types, and additional ECM coating (Figure 1B) 28. Oliver's team utilized a Pt-TPTBPF/polystyrene optical sensing material for the heart-on-a-chip oxygen sensor. The material was pre-printed on a polyethylene terephthalate (PET) substrate via a micro-dispensing technique and integrated beneath the tissue chamber during chip fabrication. This configuration enabled non-invasive, *in situ*, real-time, stable, and scalable oxygen monitoring 29. Wang et al. developed a non-invasive oxygen sensor for OoCs by fabricating a PtTFPP-doped PDMS film and integrating it via spin-coating and cutting, which eliminated the need for electrodes/wires, prevented environmental interference, and provided real-time local oxygen monitoring for enhanced data accuracy (Figure 1C) 30. The oxygen-sensing nanoparticles (NPs) was drop-cast onto the PDMS surface to form sensor spots, followed by a second layer after drying, yielding a drift of less than 0.2 % over 72 h. These integrated probes enable truly *in situ*, zero-consumption, and zero-dead-volume monitoring, and for the first time, provide micron-scale absolute oxygen tension measurements under cyclic 10 % mechanical stimulation (Figure 1D) 31; this strategy involved regulating the oxygen permeability of the chip material and utilizing cellular respiration to establish a hypoxic microenvironment (Figure 1E) 32.

Photonic biosensors constitute another important class of optical sensing platforms. Their core relies on the synergistic action of specific optical structural materials (e.g., silicon-based photonic crystals, ring resonators) and biorecognition elements (antibodies, nucleic acid aptamers, etc.). When target biomolecules bind to the recognition elements on the sensor surface, they induce changes in optical properties such as the local refractive index. These changes are then detected by the highly sensitive optical material substrate as resonance wavelength shifts or intensity variations 33. A complementary cetal oxide semiconductor (CMOS)-compatible silicon nitride micro-ring resonator was embedded beneath the pulmonary barrier. Using label-free refractive index sensing, it enabled real-time resolution of inflammatory protein secretion and barrier leakage every 19 seconds. The 28-channel parallel near-cell monitoring system quantitatively reported cytokine release from inflamed epithelium immediately after LPS stimulation (Figure 1F) 34. This fully demonstrates the powerful capability of silicon-based optical material platforms for sensing at complex biological interfaces.

The future development of optical sensors in OoCs will increasingly rely on breakthroughs in novel optical materials. Potential advancements include developing phosphorescent materials with higher photostability and lower oxygen permeability interference; photonic crystal/resonator materials possessing ultra-high-quality factors and lower detection limits; and intelligent optical composite materials capable of integrating multimodal sensing functions. Innovation in these materials serves as the key driver for enhancing sensor performance and advancing OoC technology towards higher biomimicry and reliability.

#### 2.1.2 Electrochemical sensors in OoCs

Electrochemical sensors enable in situ quantification of metabolic fluxes, ion concentrations, and transepithelial/transendothelial electrical resistance (TEER), thereby precisely resolving biochemical dynamics and barrier integrity 35. Electrophysiological sensors, primarily microelectrode arrays, simultaneously capture functional phenotypes of excitable tissues, including cardiac field potentials, neuronal action potential sequences, and conduction velocities 36. They also monitor cell adhesion, morphology, and viability via multi-frequency impedance measurements. These two modalities operate in a complementary manner, establishing an online, label-free, and multi-parameter characterization platform for OoCs.

**Figure 2:**
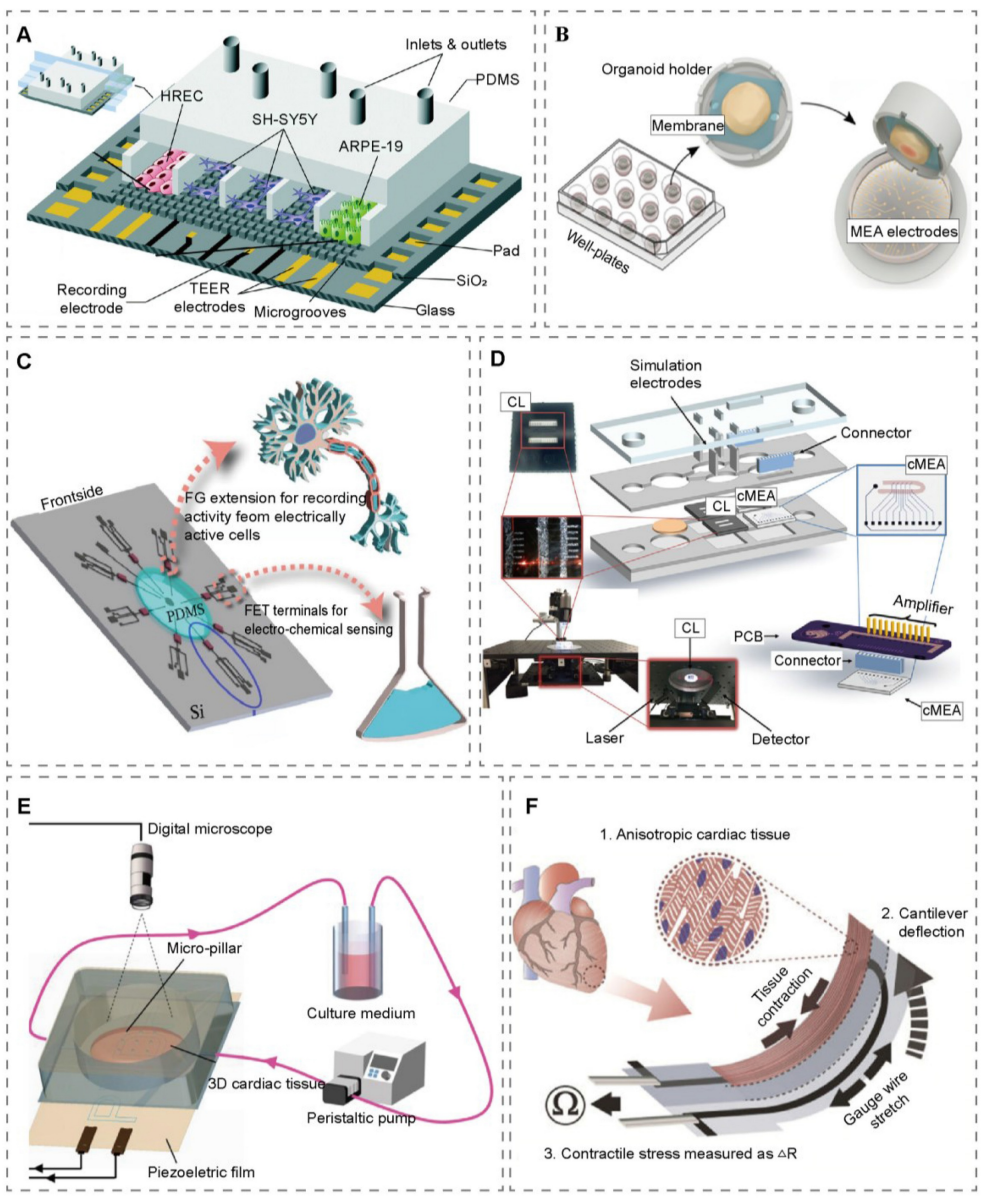
** Representative examples of electrochemical and mechanical sensors implementation in OoCs.** (A) A blood-retinal barrier-on-a-chip with crisscross microgrooves and electrophysiological electrodes. Adapted with permission from 43, copyright 2017, Royal Society of Chemistry. (B) A human spinal organoid-on-a-chip device with plug-and-play MEA electrodes. Adapted with permission from 55, copyright 2021, American Chemical Society. (C) An OoC device incorporating a multi-modal FG-FET-based sensor. Adapted with permission from 62, copyright 2023, Springer Nature. (D) A human-on-a-chip system that integrates a silicon cantilever chip for measuring mechanical and electrical functional activity. Adapted with permission from 69, copyright 2018, Wiley. (E) A heart-on-a-chip platform integrating both an image processing system and a piezoelectric sensing system. Adapted with permission from 70, copyright 2020, Elsevier. (F) A fully 3D-printed heart-on-a-chip platform instrumented with multilayer cantilevers. Adapted with permission from 71, copyright 2017, Springer Nature.

For evaluating cellular barrier integrity, electrochemical impedance spectroscopy (EIS) and TEER measurements have become the preferred non-invasive, label-free methods 37, 38. Real-time TEER monitoring effectively assesses the health status of cell layers, the formation and disruption of tight junctions, and the impact of drugs on barrier function. Traditionally, transwell systems and OoCs relied on permeability assays using fluorescent tracers to evaluate monolayer barrier function. However, these methods typically require cumbersome manual sampling, complex optical instrumentation, and offline sample processing 39, 40. Jeremy et al. innovatively replaced traditional fluorescent tracers with electroactive tracers, assessing barrier function by monitoring their diffusion across the cellular monolayer 39. They further demonstrated in a 3D hydrogel-based microfluidic vascular model that this electrochemical permeability assay maintained the robustness comparable to fluorescence methods while being easier to integrate with OoCs 41. Kosuke et al. combined electrochemical tracers with patterned electrodes to develop a novel method for electrochemical imaging of endothelial permeability using a large-scale integrated device. This method eliminated the need for independent channels, allowing electrode arrays to acquire local information; its electrodes were typically fabricated using photolithography to create gold or platinum microelectrode arrays on glass or silicon substrates. However, current systems suffer from manual operation errors and insufficient precision, necessitating future improvements in culture systems and model configurations for enhanced reliability 42. Jose employed micro-nanofabrication to construct a multi-chamber microfluidic device that integrates TEER electrodes for continuous monitoring. By simulating the blood-retinal barrier (BRB), they validated a method for placing electrodes on both sides of the cell monolayer substrate to enable real-time TEER measurement. This setup provides a more accurate assessment of cellular behavior than static models (Figure 2A) 43. The emergence of spatial transepithelial electrical resistance (S-TEER) technology further expanded TEER applications, allowing local permeability measurements in specific regions of microfluidic chips. Noa developed an S-TEER OoC system incorporating two pairs of movable miniature stainless steel electrodes, enabling resistance measurements at arbitrary locations on the OoCs. This breakthrough overcame the limited spatial resolution of traditional TEER systems, allowed precise measurements before complete cell confluence, and effectively reduced time and cost 44.

EIS reveals the electrical properties of cell layers by applying a small-amplitude AC signal to the system and analyzing its impedance response. This technique not only provides key electrical parameters such as cell layer capacitance alongside TEER measurements but also enables a more comprehensive assessment of cellular barrier integrity 45. A developed an OoC system with an embedded porous membrane impedance biosensor array, enabling non-invasive monitoring of placental barrier integrity. Its results showed high correlation with standard offline activity assays and ROS measurements 46. Mermoud integrated a micro-impedance tomography (MITO) system into a lung chip platform. This system monitored epithelial barrier integrity in real-time within 1 mm of the electrodes and successfully tracked changes in alveolar epithelial resistance during barrier permeabilization 47. Nicolas leveraged impedance spectroscopy for electrical measurements in developing the OrganoTEER instrument, which allows TEER measurements within the OrganoPlate platform. This method enables high-throughput, real-time, and non-invasive monitoring of epithelial tubules in up to 40 independent microfluidic chips, eliminating interference from artificial membranes 48.

The ability to monitor key physical and functional parameters of cardiac tissues, specifically electrophysiology and contractility, in real time and in situ is therefore critical for heart-on-a-chip 49. Zhang et al. fabricated a novel heart-on-a-chip platform integrated with soft conductive hydrogel columns. These columns are designed to concurrently monitor the contractile forces and electrical activity of 3D cardiac tissues. The acquired signals reveal a well-defined cardiac field potential profile, comprising a depolarization spike and subsequent repolarization phases, whoese characteristics are directly comparable to a clinical electrocardiogram (ECG) 50. Visone et al. has developed the μHeart platform, which integrates microelectrode channel guidance technology. This system generates functional heart tissue under biomechanical conditions and allows precise localization and monitoring of electrophysiological signals without compromising cell viability 51. Another study developed a heart-on-a-chip system integrating intracellular (Pt nanopillar arrays) and extracellular (planar electrodes) bioelectronic sensors. The extracellular devices recorded stable signals with high device yield, continuously monitoring heart rate and wavefront propagation, while the intracellular devices provided more accurate action potential recordings 52. This system precisely monitored cardiac electrophysiological changes under acute hypoxia, revealing dynamic cellular responses to oxygen deprivation. Its multiplexing capability represented a significant advance in understanding hypoxic electrophysiological responses. Li et al. constructed “cyborg organoids”, an OoC system capable of migrating and growing with cells. This system achieved the 3D implantation and distribution of nano-electronic devices within organoids. Using a 14-channel recording system, they conducted long-term, multi-channel, and tissue-scale electrophysiological recordings. Their study systematically tracked the evolution, propagation, and synchronization of spontaneous dynamics in human cardiac organoids throughout organogenesis 53.

Multi-electrode arrays (MEAs) offer advantages in electrophysiological detection. Compared to traditional patch clamp techniques, MEAs can non-invasively record electrical activity from multiple cells simultaneously, cause minimal cell damage, are suitable for long-term stable monitoring, and capture more comprehensive electrophysiological information from cell populations, providing unique advantages in throughput 54. In a study modeling the human nociceptive circuitry, an MEA system was incorporated into a spinal organoid-on-a-chip platform. This design facilitated plug-and-play electrophysiological recording by maintaining the organoids in a non-adherent state over the electrodes, which preserved electrode functionality and signal fidelity (Figure 2B) 55. Wu et al. utilized the taste organoids-on-a-chip system to simulate biological taste perception *in vitro*. In this system, MEA functioned as taste axons, receiving taste information by recording extracellular potentials of the taste organoid-on-a-chip in real time 56. Combined MEA and micro-MEA (m-MEA) to detect electrical activity in islet-on-a-chip. They also integrated a field-programmable gate array (FPGA) to enable real-time online analysis with microsecond-level latency. This approach precisely distinguished the electrophysiological activity of islet cells under different glucose concentrations, offering an innovative solution for type I diabetes research and insulin demand monitoring 57.

Field-effect transistor (FET) sensors operate based on the field-effect principle, where the conductivity of a semiconductor channel is modulated by external stimuli (e.g., biomolecules, chemical species, or light) perceived at the gate region. By transducing these interactions into measurable changes in channel conductance, FET sensors enable qualitative and quantitative detection of target analytes. They exhibit several inherent advantages, including low operating voltage, compatibility with aqueous microenvironments, and facile miniaturization, making them particularly suitable for integration into OoCs 58. Among them, graphene-based field-effect transistors (GFETs) exhibit outstanding potential, benefiting from the high carrier mobility and excellent electrical properties inherent to their two-dimensional structure 59. In 3D self-rolled biosensor arrays, GFETs utilize patterned SU-8 layers to achieve selective passivation of the sensing region, ensuring biocompatibility and enabling stable long-term monitoring. When integrated with continuous microfluidic perfusion, this configuration effectively minimizes biofouling and environmental interference. As a result, GFETs can reliably record field potentials from cardiac spheroids within complex 3D architectures 60. Furthermore, in a 3D middle ear/cochlea-on-a-chip model of the round window membrane-cochlea interface, GFETs have been successfully applied for label-free, real-time monitoring of ototoxin-induced pro-inflammatory cytokines 61. Floating-gate field-effect transistors (FG-FETs) demonstrate exceptional multi-functional integration. Effective passivation is achieved by coating the extended floating gate with a polyimide layer. This, combined with biocompatible PDMS membranes, provides a safe interface for neuronal cultures. The unique floating-gate design utilizes a capacitive coupling mechanism, which significantly suppresses signal drift caused by interfacial charge fluctuations. A notable innovation is their electrode design: a control gate functions as a pseudo-reference electrode. This simplifies the system by eliminating the need for bulky conventional reference electrodes. It allows simultaneous monitoring of pH and neuro-electrical activity, greatly enhancing system integration and reliability (Figure 2C) 62. Organic electrochemical transistors (OECTs) offer advanced solutions for long-term monitoring in dynamic physiological settings. The OECT channel material, Poly (3,4-ethylene-dioxythiophene): poly (styrene sulfonic acid) (PEDOT: PSS), exhibits hydrogel-like properties. These properties promote cell adhesion and ensure efficient ion-to-electron coupling. An optimized AC driving mode minimizes signal drift by controlling doping depth. The gate can be functionalized for specific detection (e.g., glucose), or the source-drain electrodes can be shorted to create controlled electroporation wounds in cells. This enables the integration of sensing and manipulation capabilities within OoCs 63.

Despite these collaborative advances in materials, devices, and system integration, which are driving FET-OoCs toward greater reliability and functionality, several challenges remain for their mature application. The long-term stability of current passivation materials in complex biofluids needs improvement, necessitating novel biomimetic or nanocomposite coatings for chronic studies 64, 65. Signal parsing is complicated by the coupling of multiple physiological parameters; decoupling these signals requires multimodal sensor arrays combined with machine learning (ML) 66. Fabrication and integration face bottlenecks due to the custom nature of current designs, highlighting the need for standardized, “sensing-first”, wafer-scale processes. Furthermore, achieving conformal contact between sensors and 3D organoids, along with the lack of industry-wide standardized performance assessment protocols, hinders their application in physiologically relevant models. These systemic challenges represent critical hurdles that must be overcome to transition FET-sensing OoC technology from the laboratory to a standardized biological tool.

#### 2.1.3 Mechanical sensors in OoCs

Mechanical sensors are key components in OoCs for monitoring tissue mechanical behavior, particularly for the real-time assessment of contractile function in parenchymal cells such as cardiomyocytes. Microcantilevers, as a common type of mechanical sensor, are integrated into OoCs via monolithic designs to enable non-invasive and highly sensitive mechanical monitoring. For instance, one study used integrated 3D stamping to form UV-crosslinked polyester elastomer microcantilevers (150 μm) directly onto the sidewalls of an AngioChip bioscaffold, making them an inseparable part of the structure. This design requires no additional electronic components and operates synergistically with the scaffold's 15 μm sidewall pores and a gravity-driven perfusion system (flow rate: 1.4 μL/min; shear stress: 1.05 dyne/cm²). When cardiomyocytes contract spontaneously or in response to pharmacological stimuli, the microcantilevers deflect. This displacement is quantified via fluorescence microscopy and ImageJ software to indirectly derive contractile force and beating patterns 67. Other studies similarly demonstrate diverse implementations of microcantilevers: AFM silicon cantilevers (737 μm×150 μm×4 μm) fabricated from silicon-on-insulator wafers utilize laser deflection and a modified Stoney equation to accurately measure cardiomyocyte contractile stress^68^; a silicon cantilever chip integrating 32 cantilevers employs laser deflection for long-term monitoring of contractile forces in human induced pluripotent stem cell (iPSC)-derived cardiomyocytes and skeletal muscle cells in human multi-organ in vitro platforms (Figure 2D) 69.

Beyond microcantilevers, other mechanical sensors are widely used in OoCs. In a heart-on-a-chip, PDMS micropost arrays physically engage with cardiac tissues, converting contractile forces into bending deformations. Equivalent contractile forces are quantified using digital microscopy and matrix laboratory (MATLAB) code, enabling multi-site monitoring and analysis of contraction synchrony. Meanwhile, polyvinylidene fluoride (PVDF) piezoelectric films, attached to the bottom of micropost arrays, transform mechanical stress into voltage signals via the piezoelectric effect, providing millisecond-level response for monitoring overall contraction strength and frequency (Figure 2E) 70. Johan et al. embedded strain sensors based on carbon black-thermoplastic polyurethane composite inks, which are integrated into cantilever structures within heart-on-a-chip via multi-material 3D printing. These sensors convert mechanical deflection into resistance signals, allowing for the *in situ* detection of cardiac tissue contractile stress (within the 1-15 kPa range) and supporting long-term drug testing (Figure 2F) 71. Flexible polyether ether ketone (PEEK) probes are a type of functional probe device with flexibility, using PEEK as the core substrate. Their key advantages lie in the excellent biocompatibility of PEEK itself, which enables long-term *in vivo* implantation and effectively reduces the body's immune rejection. Meanwhile, the flexible design can adapt to the dynamic deformation of biological tissues, further reducing the risk of mechanical damage. Veniamin et al. used flexible PEEK probes to serve as mechanical sensors, combined with floating microelectrodes in the I-Wire heart-on-a-chip to simultaneously monitor the contractile force and electrophysiological function of 3D cardiac tissues. Force values are derived through image analysis and calibrated linear stiffness coefficients, making the system suitable for drug screening applications 72.

These mechanical sensors share common features such as structural compatibility, non-invasiveness, and functional integration, enabling synergistic operation with OoCs' perfusion systems for real-time, highly sensitive mechanical monitoring. Despite these advantages and their core role in assessing mechanical function, several technical defects hinder their standardized application. A significant limitation is the reliance of many high-sensitivity optical sensing strategies on sophisticated and costly readout systems, which restricts long-term, high-throughput use. While non-optical alternatives like embedded electronic sensors exist, they often require custom interfaces and suffer from challenges in long-term signal stability and hysteresis. Furthermore, the accurate conversion of raw sensor signals into absolute biomechanical parameters remains problematic, as the underlying analytical models are sensitive to uncertainties in material properties and cell-material interactions, potentially introducing significant quantification errors. Finally, achieving truly seamless integration often involves a trade-off, where monolithic designs may limit sensor replaceability, and the co-integration of multiple functionalities can lead to signal crosstalk or manufacturing complexities. Addressing these defects is crucial for advancing OoCs from proof-of-concept tools toward robust and standardized platforms for biomedical research.

#### 2.1.4 Multi-sensors in OoCs

OoC technology derives its core advantage from its ability to simulate the complex microenvironment of human organs. Multi-sensor integration technology achieves real-time, *in situ*, and parallel monitoring of multiple critical parameters within the cellular microenvironment (such as barrier integrity, metabolite concentration, ion activity, electrophysiological signals, pH, oxygen tension, etc.) by synergistically integrating various physical, chemical, and biological sensors at the material level onto a single microfluidic platform, as summarized in Table 1 73. This multimodal sensing strategy, based on advanced functional materials, provides unprecedented comprehensive characterization for gaining deeper insights into tissue behavior, dynamic cell function, drug effects, and responses to environmental perturbations. It serves as the key enabling technology propelling OoCs from model construction towards precise prediction.

**Figure 3:**
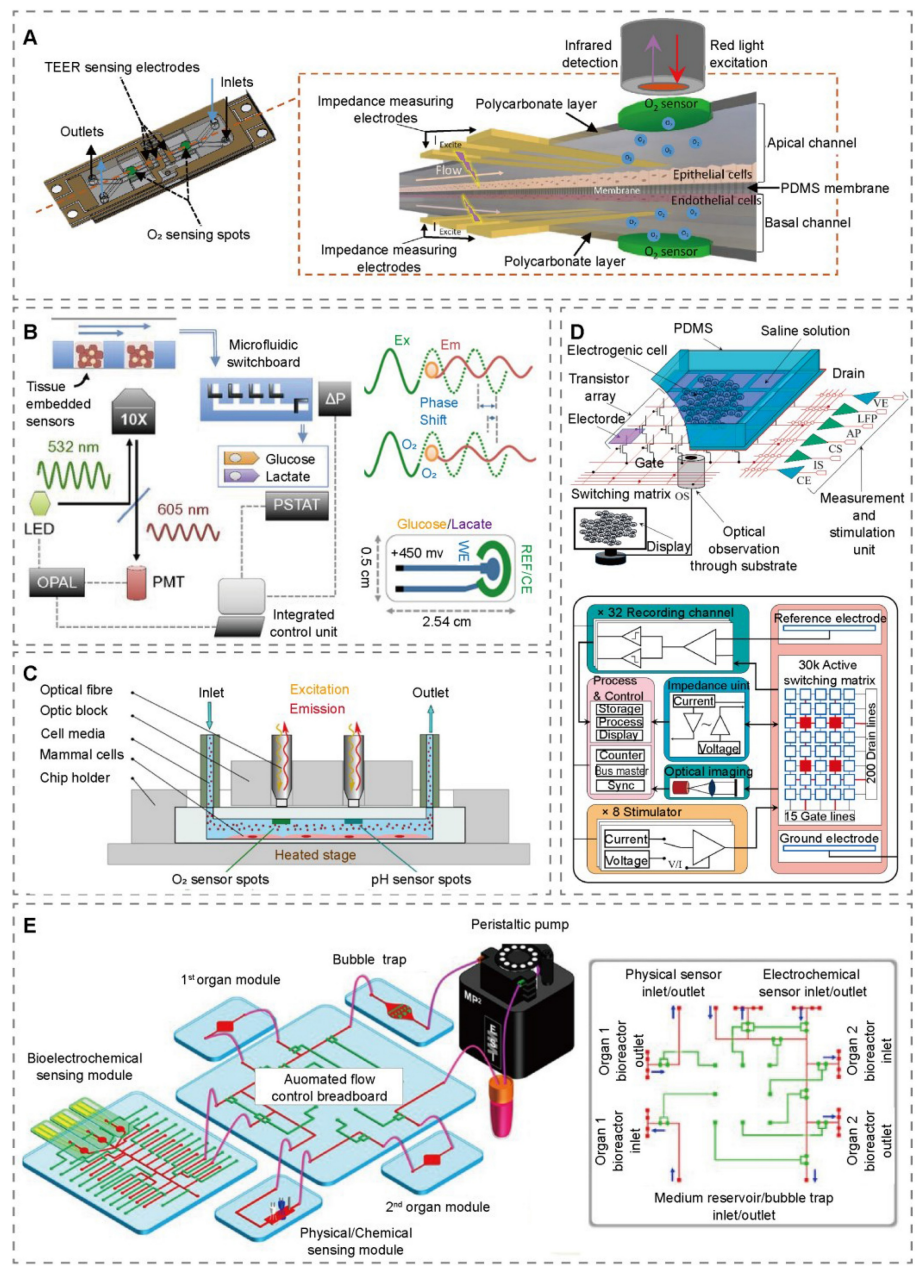
** Representative examples of multi-sensor implementation in OoCs.** (A) A dual-channel chip with integrated TEER and oxygen sensors. Adapted with permission from 74, copyright 2024, Elsevier. (B) A liver-on-a-chip device with dual-frequency phase modulation of tissue-embedded phosphorescent microprobes and amperometric glucose and lactate sensors. Adapted with permission from 75, copyright 2016, National Academy of Sciences. (C) A thermoplastic microfluidic device integrating miniaturized optical pH and O_2_ sensors. Adapted with permission from 76, copyright 2021, Elsevier. (D) An optically TFT array biosensor chip for neuronal ensemble investigation. Adapted with permission from 80, copyright 2020, Elsevier. (E) An integrated OoC platform with optical, physical sensors, and electrochemical biosensors. Adapted with permission from 79, copyright 2017, National Academy of Sciences.

Real-time monitoring of cellular metabolic activity is central to evaluating OoCs' function. Multi-parameter sensing overcomes the limitations of single indicators, providing a more comprehensive picture of metabolic status and drug biological effects. Zohreh et al. developed siChip, a representative example of multi-sensor integration. By incorporating TEER sensors, oxygen sensors, pH sensors, and metabolic activity sensors, this platform enables real-time monitoring of key parameters such as barrier function, oxygen concentration, and metabolic activity. The design allows for the simulation of specific physiological conditions while analyzing dynamic cellular responses to chemical stimuli (Figure 3A) 74. For the measurement of glucose, lactate, and oxygen in a liver-on-a-chip bioreactor under physiological conditions, an integrated phosphorescent microprobe and electrochemical sensor technology has been developed. This system detected metabolic changes and ATP redistribution following drug treatment, enabling the assessment of mitochondrial dysfunction at pharmacologically relevant concentrations (Figure 3B) 75. Bernhard utilized PtTPTBPF-stained PtBS particles for oxygen sensing and a pH sensor consisting of OHC12 and silanized Egyptian blue microcrystalline powder. These sensors were embedded in hydromed D4 hydrogel matrices, allowing real-time monitoring of oxygen and pH levels in the cell culture environment through specific light excitation and detection (Figure 3C) 76.

Continuous *in situ* measurement is crucial for accurately capturing microenvironment dynamics and long-term organ responses to drugs, relying on stable, biocompatible, and integrable sensing materials alongside microfabrication techniques. A gut-on-a-chip device integrating label-free sensors with MEMS has been developed for TEER measurement. This system not only enabled real-time, non-invasive monitoring of TEER changes but also quantitatively analyzed mercury ion absorption 77. Maoz et al. was the first to integrate TEER and MEA into a heart-on-a-chip system. TEER measured tissue barrier integrity and permeability, while MEA evaluated the function of excitable cells. This system allowed simultaneous assessment of cellular electrical activity and barrier function 78. Researchers have developed an automated modular design platform that integrates label-free electrochemical immunosensors for *in situ* continuous monitoring of soluble biomarkers secreted by organoids. By employing specific functionalization and regeneration processes, the platform enhances detection sensitivity and repeatability. Additionally, it integrates optical pH sensors, oxygen sensors, and temperature probes to monitor microenvironmental parameters. A miniature microscope is also incorporated for real-time observation of organoid morphological changes (Figure 3E) 79.

Moving beyond traditional sensors, novel electronic materials and bioprinting technologies open new pathways for OoCs sensing integration. Thin-film transistor (TFT) is expected to play an increasingly significant role in OoCs detection. Faruk developed a TFT-based array biosensor chip featuring six operational modes, including extracellular voltage recording, electrical and chemical stimulation, and optical imaging. This chip enables a multi-faceted approach to studying neuronal networks. The ability to monitor complex neuronal activity is greatly enhanced by its high electrode fill factor of 97.5%, high temporal resolution of 10ms, and optical transparency of over 90% (Figure 3D) 80. Bioprinting enables the precise and reproducible integration of organoids into modular perfusion devices. Based on this, Aleksander has developed a triple tissue OoCs for liver, heart, and lung that integrates three electrochemical detection technologies: electrochemical impedance, TEER, and short-circuit current (Isc). The electrochemical impedance sensor enables the real-time, continuous, and multi-metric detection of various soluble biomarkers such as albumin, α-GST, and creatine kinase secreted by tissue constructs to assess tissue function and drug response. The TEER assay and Isc assay are mainly used in the lung microarray module to assess the integrity of lung organoids, monitor the activity of ion channels, and investigate the effects of drugs on lung tissue 81.

Through multimodal integration and material-driven innovation, biosensors enable real-time, *in situ*, and parallel monitoring of critical parameters within complex microenvironments. These parameters include barrier integrity, metabolite dynamics, electrophysiological signals, oxygen tension, and pH. Sensing technologies based on advanced functional materials, such as phosphorescent probes, flexible electrodes, smart hydrogels, and biocompatible nanocomposites, overcome the destructive nature and low spatiotemporal resolution limitations of traditional offline methods. This significantly enhances the biomimetic fidelity of OoCs. However, advancing sensor performance further still confronts critical challenges. Key among these are the need for novel micro/nano-fabrication materials and heterogeneous integration technologies to achieve miniaturization and integration; the reliance on biocompatible, anti-fouling interfacial materials and stable transducer materials to ensure long-term operational stability; and the demanding requirements for the mechanical properties of flexible/implantable materials coupled with precise fluid dynamics design to ensure compatibility within the OoCs microfluidic environment 18. Overcoming these hurdles fundamentally requires innovation in advanced functional materials, such as multifunctional nanocomposites, bioinspired interfacial materials, and highly stable sensing materials.

### 2.2 Fluorescent probes in OoCs

In OoCs research, the real-time, *in situ* monitoring of the cellular microenvironment is essential. Fluorescent probes, which emit light across the ultraviolet to near-infrared spectrum, have emerged as a core analytical technology in this field. Their utility stems from the unique photophysical properties of their fluorophores and the highly designable nature of their molecular or nano-scale structures 83. Compared to traditional methods requiring chip destruction or microfluidic interruption, non-invasive optical signal acquisition using fluorescent probes enables *in situ* target molecule capture. This maintains chip integrity while significantly reducing data distortion risks from sampling interference. Fluorescent probes, leveraging signal amplification via nanomaterials or molecular recognition mechanisms, can detect target molecules at concentrations as low as pM levels 84. The deep tissue penetration and low biotoxicity of nanoprobes further ensure the feasibility of long-term live imaging in 3D organoids 85. Additionally, multimodal integration of fluorescent probes with technologies such as electrophysiological recording and microfluidic pressure sensing enables the construction of multi-scale analysis platforms from molecular events to organ functions.

#### 2.2.1 Molecular fluorescent probes in OoCs

Molecular fluorescent probes typically comprise three key components: a recognition group, a signaling fluorophore, and often a linker. This structure enables the conversion of target binding or chemical reactions into detectable fluorescence changes, such as intensity variation, spectral shift, or lifetime alteration. Small-molecular dyes are extensively utilized in fluorescence imaging due to their excellent biocompatibility and flexible chemical modifiability 86. However, their utility in long-term imaging is often constrained by photobleaching and limited photostability 87. Based on their activation mechanisms, these probes are classified into three categories: chemically responsive, biomolecular responsive, and physically responsive 88, 89.

**Figure 4:**
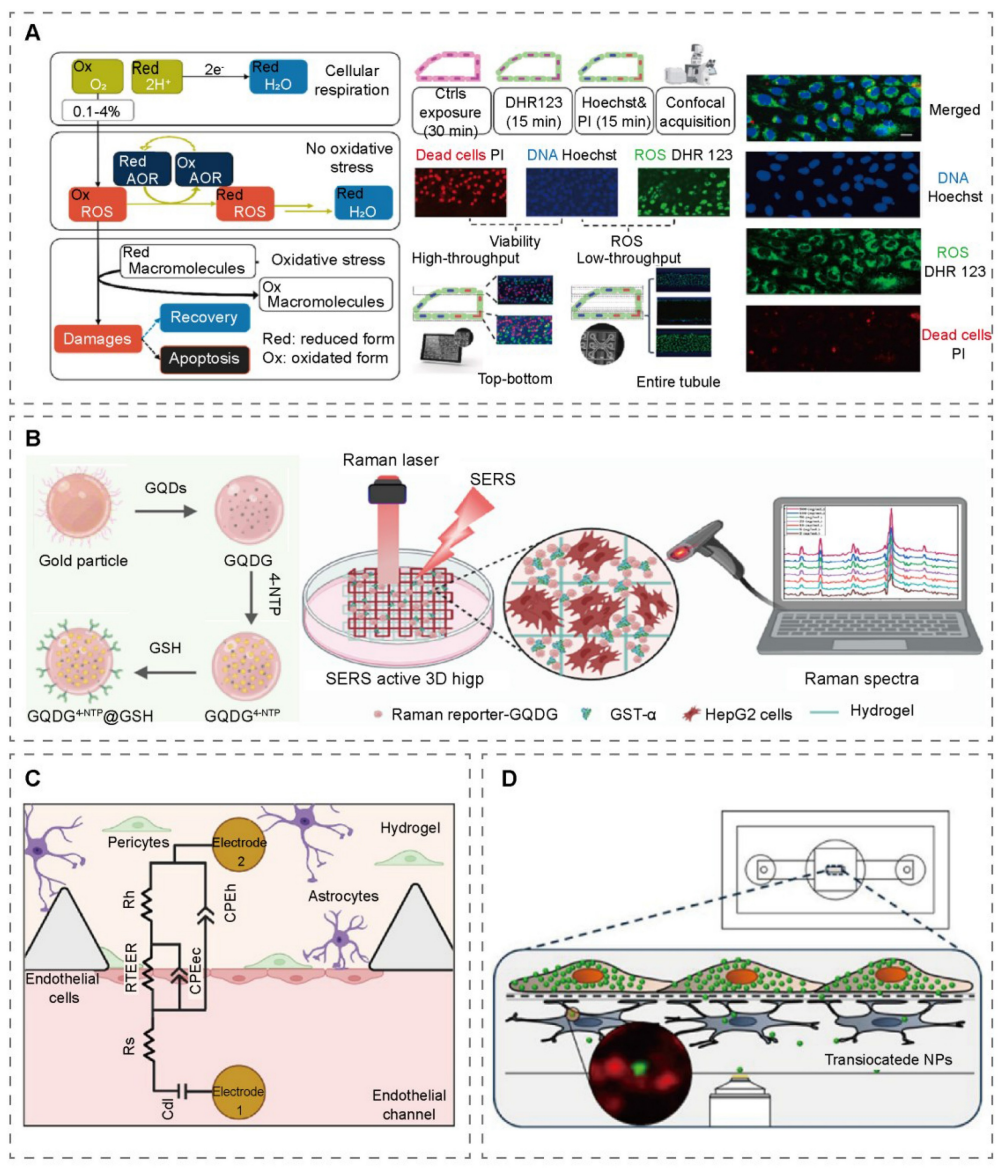
** Representative examples of fluorescent probes implementation in OoCs.** (A). A vasculature-on-a-chip for the quantification of intracellular ROS and cell viability. Adapted with permission from 93, copyright 2022, Elsevier. (B) A 3D-bioprinted liver-on-a-chip implanted in graphene-based plasmonic sensors Adapted with permission from 106, copyright 2024, American Chemical Society. (C) BBB-on-a-chip platform labeled with the GNR-PEG-Ang2/D1-A647 probe. Adapted with permission from 110, copyright 2023, Springer Nature. (D) BBB-on-a-chip labeled with ApoE-SiO₂ probes for live-cell imaging. Adapted with permission from 112, copyright 2020, American Chemical Society.

Chemically responsive probes undergo fluorescence changes via ion chelation or redox reactions. Fluo-4 acetoxymethyl ester (Fluo-4 AM), for example, enables real-time recording of calcium transients in single cells inside microfluidic chips with millisecond resolution 90, 91. Probes such as 2',7'-dichlorofluorescin diacetate (DCFH-DA) and 4-amino-5-methylamino-2',7'-dichlorofluorescin diacetate (DAF-FM DA), widely used for reactive oxygen and nitrogen species (ROS/RNS) detection (Figure 4A), are now available as standardized commercial assay kits 92, 93. Biomolecular-responsive probes are activated through interactions with biomacromolecules such as enzymes or nucleic acids. 5-chloromethylfluorescein diacetate (CMFDA), activated by intracellular esterases and glutathione, facilitates long-term tracking of multicellular dynamics in OoCs 94. Calcein-AM/propidium iodide (PI) assays are routinely applied for viability/cytotoxicity testing 95, while cytochrome P450 probe substrates allow dynamic assessment of metabolic enzyme activity in liver-on-a-chip 96. Physically responsive probes exhibit fluorescence changes in response to physical parameters. FRET-based sensors can monitor molecular distance changes in real time; for instance, a caspase-3 FRET sensor visualized apoptosis in endothelial cells under flow 97. FITC-dextran, valued for its water solubility and stability, serves as a standard probe for assessing barrier permeability (e.g., in BBB, placental, and intestinal models) and has also been utilized in tumor-on-a-chip to quantify drug penetration and interstitial pressure 98, 99.

#### 2.2.2 Nanoparticle fluorescent probes in OoCs

Nanoparticle fluorescent probes are multifunctional composite systems constructed based on nanomaterials. Their composition typically includes a nanocarrier core, a fluorescent unit, targeting modifications, and a functional coating. Due to their unique physicochemical properties, nanomaterials are highly suitable for biological monitoring and imaging 100. In contrast to traditional organic dyes, nanoparticle fluorescent probes provide exceptionally tunable excitation and emission profiles, which can be engineered by adjusting their physical size and elemental makeup. This rational design capability underpins their outstanding optical performance, characterized by high signal intensity, robust photostability, extended lifetime, and favorable biocompatibility 101. It can be classified based on its material composition into categories such as quantum dots (QDs), upconversion nanoparticles (UCNPs), gold nanorods (AuNRs), and silica nanoparticles (SiNPs) probes 102.

QDs are semiconductor nanocrystals (2-10 nm) whose fluorescence properties are dominated by quantum confinement effects. By tuning their size and composition, QDs can achieve tunable emission spectra ranging from ultraviolet to infrared. Due to their high brightness, photobleaching resistance, and multi-color labeling capability, QD-based fluorescent probes have become essential tools for OoCs characterization 103. In a biomimetic alveolus-epithelium-on-a-chip study, ZnO-QDs were encapsulated in human serum albumin (HSA) NPs approximately 60 nm in diameter. The resulting HSA-ZnO NPs exhibited excellent biocompatibility, pH-responsive targeted release, and efficient cellular internalization 104. Wu et al. incorporated cadmium selenide (CdSe) /ZnS core-shell quantum dots into thermoplastic elastomers (TPE) to fabricate TPE/QD nanocomposites. This material enables long-term electrical stimulation and simultaneous *in situ* assessment of cardiac contractility 105. GQDs-AuNPs are composite nanomaterials formed by graphene quantum dots (GQDs) and AuNP svia chemical bonding or physical adsorption. This system integrates the fluorescent properties and surface modification flexibility of GQDs with the plasmonic resonance effect of AuNPs. Zhu et al. incorporated GQDs into a hydrogel matrix within a liver-on-a-chip bioprinted in 3D, achieving *in situ* quantification of glutathione S-transferase alpha (GST-α) secretion by liver cells. The detection system exhibited a dynamic linear range of 20-500 ng/mL with high specificity and anti-interference capability (Figure 4B) 106. UCNPs are a class of inorganic nanomaterials doped with rare-earth elements that absorb Low-energy near-infrared (NIR) light and emit higher-energy visible or ultraviolet light, effectively reducing autofluorescence interference 107. Wimalachandra et al. developed a novel UCNP probe for characterizing the tumor-vascular interface in OoCs. The probe features mesoporous silica-coated UCNPs co-functionalized with folic acid (FA) and the secondary lymphoid tissue chemokine (CCL21). Upon 980 nm excitation, these CCL21-FA-UCNPs exhibited distinct fluorescence emissions at 410, 543, and 658 nm. Furthermore, the probe demonstrated excellent biocompatibility, showing low cytotoxicity, a minimal hemolysis rate, and good blood compatibility 108.

AuNRs are anisotropic metallic nanomaterials with multimodal functions. Their longitudinal surface plasmon resonance (SPR) absorption peaks, typically in the near-infrared region (650-900 nm), can be precisely tuned by adjusting their aspect ratio. Since their optical signals are highly sensitive to the refractive index of the surrounding medium, AuNRs are frequently employed as surface plasmon resonance probes 109. AuNRs were conjugated with the Alexa647 fluorophore to construct a traceable probe, designated as GNR-PEG-Ang2/D1-A647. This probe enabled real-time observation of AuNRs distribution between the cerebral endothelial channel and the central chamber in a BBB-on-a-chip model, thereby providing direct evidence for the quantitative assessment of the BBB-penetrating capacity of the AuNR-based nanomedicine (Figure 4C) 110.

SiNPs probes are composite nanoprobes that use SiO₂ as a carrier, incorporating fluorescent substances through physical encapsulation or chemical bonding. The SiO₂ shell isolates oxygen, free radicals, and biological enzymes, significantly extending the lifespan of fluorescent dyes while offering high stability, ease of functionalization, and modifiable surface properties 111. Hudecz et al. developed an *in vitro* BBB model (μSiM-BBB) using fluorescent probes, including 40 nm PS-COOH and 100 nm PS-COOH polystyrene nanoparticles, as well as FITC-labeled apolipoprotein e-modified 100 nm SiO₂ (ApoE-SiO₂) nanoparticles. By employing high-resolution fluorescence imaging techniques, researchers investigated the interaction between these fluorescent nanoprobes and the BBB, focusing on subcellular localization of nanoparticles within endothelial cells, intracellular transport processes, and translocation events across the BBB into the astrocyte layer (Figure 4D) 112. By implementing stepwise functionalization, nanoparticles can ensure independent yet synergistic functionality of different functional modules. Ag@SiO₂-FITC NPs are composite nanoprobes composed of a silver nanoparticle (AgNPs) core, a SiO₂ shell, and covalently bound FITC. This structure integrates the plasmonic effects of the silver core, the protective and functional capabilities of the silica shell, and the fluorescent properties of FITC, enabling microenvironmental pH monitoring through the pH sensitivity of FITC. Researchers immobilized these nanoprobes covalently onto the glass substrate of a microfluidic channel to form a sensing surface for real-time, ratiometric imaging of biofilms formed by the oral bacterium Streptococcus salivarius. This approach allowed the investigation of biofilm pH dynamics under various chemical (glucose concentration changes) and hydrodynamic (shear stress induced by different flow rates) stimuli 113.

Fluorescent probes, with their molecular-level spatial resolution, millisecond-level dynamic response capability, and multi-parameter synchronous monitoring characteristics, have become core tools for* in situ* analysis of the microenvironment in OoCs, advancing real-time cross-scale observation from ion fluctuations to tissue functions. The key to future breakthroughs lies in: developing probes with enhanced photostability to overcome long-term imaging bottlenecks; creating orthogonally multi-encoded probes to achieve decoupling of ultra-complex biological signals; deeply integrating AI algorithms to improve *in situ* quantification accuracy; and developing biomimetic micro-interface integration technologies to coordinate probe functions with the dynamic physiological microenvironment of OoCs, ultimately driving this technology from static observation to dynamic regulation.

### 2.3 Image visual recognition in OoCs

Advancements in microscopy have greatly facilitated research on cell-cell interactions, cellular behaviors, and dynamic changes within the microenvironment. Using confocal microscopy, high-content imaging systems, optical coherence tomography (OCT), wide-field fluorescence microscopy, and high-speed imaging, researchers can visualize cellular dynamics, intercellular interactions, and microenvironmental changes within OoCs in real time, providing high-resolution visualization tools for disease mechanism studies and drug screening.

Transmission light microscopy, one of the earliest developed microscopic techniques, utilizes contrast formed by light passing through a sample and is widely used for observing the microstructures of cells and materials. Despite the emergence of advanced microscopy techniques, transmission light microscopy remains valuable in fundamental research due to its simplicity and ease of sample preparation. For instance, Alexandra et al. used transmission light microscopy in combination with a fusion touch syringe pump to apply different flow rates to colon-on-a-chip, generating side-view images and dynamic videos to reveal the response of the colonic mucus layer to varying shear stresses 114. OCT reconstructs 2D/3D images of samples by detecting optical interference signals, making it widely applicable in vascular and tissue structure studies. Yusuf et al. employed OCT imaging to assess hydrogel matrices and the 3D microvascular structures formed within an outer blood-retinal barrier model, enabling evaluation of vascular barrier integrity 115. SPM allows direct surface scanning of samples to obtain spatial information on *in situ* chemical and biological processes. Nashimoto et al. utilized scanning probe microscopy (SPM) to overcome the challenges of high-resolution imaging and real-time analysis in a vasculature-on-a-chip posed by traditional optical tools. The nanoscale imaging capability of scanning ion conductance microscopy (SICM) surpasses the diffraction limit, while scanning electrochemical microscopy (SECM) and SICM measurements do not compromise cellular integrity, enabling real-time monitoring of cellular functions 116.

Furthermore, atomic force microscopy (AFM), a high-resolution scanning probe technology, also plays a crucial role in the characterization of OoCs. Guido et al. combined MEA with AFM technology to simultaneously record the electrical activity and mechanical contractility of cells in a cardiac-on-a-chip, providing an innovative approach for cardiac disease modeling and drug screening 117. Similarly, Ignasi et al. developed a novel PDMS chip utilizing AFM to probe the nonlinear mechanical properties of tissue samples 118. As a non-destructive analytical technique based on the interaction between light and matter, Raman spectroscopy can obtain molecular vibrational and chemical information by detecting changes in scattered light frequency 119. Manjot et al. integrated Raman spectroscopy with a 3D-printed conformal microfluidic device to achieve non-invasive, real-time biomarker detection and isolation 120. Calogiuri et al. used Raman spectroscopy, which provides a non-invasive, real-time, and label-free analysis modality, to monitor structural and biochemical changes in the tight junctions (TJs) of a Caco-2 intestinal epithelium-on-a-chip 121.

### 2.4 Application of AI in the detection technology of OoCs

AI, particularly ML and deep learning (DL), is revolutionizing the analysis of complex data by enabling the identification of patterns and predictive modeling beyond human capability. In the context of OoCs, which generate vast, multimodal datasets, AI serves as an indispensable analytical tool. Its applications span three primary domains: First, in imaging modalities processing, where AI algorithms automate the quantification of cellular structures, track dynamic behaviors, and classify phenotypes from high-content microscopy data. Second, AI extends to deciphering non-imaging modalities, such as interpreting spectroscopic signals (e.g., Raman) and other sensor data for label-free biochemical assessment. Finally, its most powerful application lies in integrative data analysis, where AI consolidates multi-parametric readouts, functional assays, and even external databases to uncover complex biological correlations and predict systemic outcomes 122, 123.

#### 2.4.1 AI-powered analysis of image modalities in OoCs

OoC technology excels at modeling complex physiology by precisely controlling the cellular microenvironment. This capability, however, generates multidimensional and high-throughput data—encompassing parameters such as fluid shear stress, molecular gradients, and cellular dynamics—which poses significant challenges for conventional analytical methods. AI, particularly DL, addresses this bottleneck by automatically extracting features and recognizing patterns from high-dimensional datasets 124. This approach not only enhances research productivity but also uncovers hidden biological insights with speed and accuracy.

Vascularization is becoming a critical physiological organ-level feature required for OoCs, yet there are no standard tools or morphological indicators to measure the performance or biological function of vascular networks in these models. James et al. were the first to apply ML to predict the biological function of vascular networks in a vascularized microphysiological system (vMPS). To predict the oxygen transport function in OoCs, they employed ML models including multiple linear regression (MLR), decision tree (DT), and random forest (RF) to analyze morphological image data of the vascular network. Among these, the random forest model demonstrated the best performance 125. In another study, they analyzed 500 images of vMPS using AI to extract morphological and oxygen transport parameters. They introduced a vascular network quality index (VNQI) using a chain neural network. This index was validated in a vascularized islet-on-a-chip, where chips with higher VNQI values demonstrated superior insulin secretion function under hypoxic challenge 126. Danial et al. used vMPS with an ultra-thin nanoporous silicon nitride membrane to simulate the human vascular microenvironment for studying leukocyte migration events. AI was employed with random forest semantic segmentation and LeNet-5-based convolutional neural network classification algorithms to analyze the spatiotemporal distribution, migration rate, and activity indicators (such as speed, persistence, and tortuosity index) of leukocytes in co-culture models. This automated analysis tool set was comparable to manual statistical data sets in terms of computational efficiency and could semi-automatically process large imaging datasets 127.

In tumor immunology research, the integration of OoCs with AI has enabled precise characterization and dynamic tracking of the immune microenvironment. For example, Zheng et al. employed a DL based on tumor-infiltrating lymphocytes (TILs) scoring, combined with a 3D tumor-stroma spheroid-on-a-chip, to efficiently screen an epigenetic modulator drug library and identify the synergistic effect of lysine-specific demethylase (1LSD1) inhibitors with immune checkpoint inhibitors 128. Similarly, Stefania and Elena, using a microfluidic platform and the unsupervised learning algorithm “Cell Hunter”, achieved real-time tracking of dendritic cells and PBMCs within the tumor microenvironment 129, 130.

In studies analyzing cellular behavior in complex microenvironments, Chen et al. applied the visual geometry group (VGG)-19 to precisely characterize dynamic processes in a lung-on-a-chip. The VGG-19 model achieved a prediction accuracy of over 90% per single image after training. With an ensemble analysis of four images, an accuracy of 99.8% was achieved 131. Similarly, Marc et al. used automated image analysis techniques to quantify vascular network features in a 3D vascular-on-a-chip, including vessel density, diameter, and branching point density 132. These studies highlight the application of AI in OoCs, not only for characterizing microscopic biological processes but also for providing crucial references for disease modeling and drug development.

DL, such as convolutional neural networks (CNNs), have demonstrated outstanding performance in image recognition and analysis. CNNs can automatically learn features from images, enabling pixel-level classification, segmentation, and object detection while overcoming the limitations of manually designed features in traditional image analysis. This capability allows for more accurate identification and quantification of biological information in images 133. Bhanu et al. utilized CNNs to achieve precise characterization and dynamic monitoring of skeletal muscle cells. By analyzing cellular images with CNNs, they identified changes at the nanometer level in cell images and inferred the functional performance of muscle cells. Furthermore, the team integrated recurrent neural networks (RNNs) and long short-term memory (LSTM) modules to predict time-series data from dynamic cellular mechanics image sequences. By combining supervised and unsupervised DL architectures, they effectively tracked dynamic changes in cellular markers concerning morphology, composition, and function 134. Additionally, by employing CNNs for the automated analysis of β-catenin nuclear translocation images in a biomimetic bone-on-a-chip, researchers achieved 99.5% accuracy in the high-throughput, intelligent assessment of osteoporosis drug efficacy 135. Chen et al. employed DL based on pre-trained CNNs, such as residual network (ResNet) and score-class activation map (CAM) feature visualization methods to analyze bright-field images of human small airway epithelial cells cultured for three days. By applying data augmentation and five-fold cross-validation for model training, they achieved a cell differentiation prediction accuracy of 89%. Moreover, the integration of an automated ciliary beating frequency calculation model improved efficiency by 90% compared to manual methods 136. Sungho et al. developed a 3D microfluidic bladder cancer-on-a-chip for culturing bladder cancer cell lines with different levels of drug resistance alongside endothelial cells. The CNN analyzed 2674 cell images generated by the OoCs, covering different levels of gemcitabine resistance in bladder cancer cells. The system effectively predicted the anticancer drug resistance of bladder cancer, with CNN achieving a prediction accuracy of up to 95.2% when using data augmentation and a learning rate decay step (initial value 0.001) 137. In the high-throughput biomimetic bone-on-a-chip built by Kyurim et al., CNN analyzed the large volume of image data obtained from bone-on-a-chip, including β-catenin, cell nuclei, and merged fluorescence images, to evaluate the effectiveness of anti-osteoporosis drugs. By assessing the intensity and nuclear translocation rates of β-catenin, the system improved the accuracy and reliability of drug efficacy evaluation, providing a new and powerful tool for bone-related disease research and drug development 138.

#### 2.4.2 AI-powered analysis of non-image modalities in OoCs

Beyond microscopic imaging, spectroscopic data from OoCs present a rich source of information for AI-driven decoding. For instance, Raman spectroscopy, a label-free technique, can monitor biochemical changes in living cells within OoCs. In a study modeling the intestinal epithelial barrier using a Caco-2 cell monolayer-on-a-chip, Raman spectra were collected from cell junction regions under different treatment conditions. To manage the high-dimensional spectral data, Principal Component Analysis was first employed to reduce complexity and identify key features associated with tight junctions, such as peaks related to E-cadherin. Subsequently, a supervised ML model, specifically a quadratic Support Vector Machine, was trained on these selected features. Using only 7 features, this model achieved 91.9% accuracy in classifying different damage states of cellular tight junctions. This demonstrates the potential of combining label-free spectroscopy with AI for precise phenotypic assessment 121. Furthermore, innovative sensing architectures can generate data formats highly amenable to AI-driven compression and reconstruction. One study employed a temporal-spatial compressed sensing (TS-CS) framework for acquiring optical signals from fibroblast micro-tissues. Although the signal source was optical, the data underwent compression in the analog domain before digitization. AI-inspired algorithms, specifically L₁-norm minimization based on DCT or DWT basis functions, were then used to reconstruct the original tissue dynamics. This approach achieved high fidelity and significantly improved data throughput and signal-to-noise distortion ratio compared to traditional methods. It highlights a synergy between advanced hardware design and computational algorithms to overcome data acquisition bottlenecks in OoCs 139.

The utility of AI in OoCs extends beyond analyzing single sensing signals. Its greater value lies in integrating multi-parametric readouts and even external databases for higher-level prediction and discovery. In a multicellular coculture array (MCA) designed for predicting drug-induced skin sensitization, each drug treatment generated five normalized quantitative cellular functional readouts. A Support Vector Machine model was used to integrate these multi-dimensional readouts, successfully correlating them with the sensitization potential of drugs. The model achieved 87.5% accuracy and 100% sensitivity, providing a powerful tool for systemic toxicity screening 140. In a more forward-looking study, an ML liver-on-a-chip was developed for safer drug formulation. This research integrated experimental data from a porcine liver-on-a-chip with large external datasets. These included over 50,000 cytochrome P450 enzyme inhibition data points from ChEMBL24 and molecular fingerprints of candidate compounds. A Random Forest ML model was trained on this heterogeneous data to predict molecules that could mitigate acetaminophen hepatotoxicity. This AI-driven approach handled conflicting data annotations and successfully identified protective excipients, such as rutin and eugenol, from FDA-approved substances. It establishes a “predict-validate” closed-loop development model 141.

AI has emerged as a powerful tool for deciphering the multimodal data generated by OoCs. Its strengths lie in automating the extraction of key features from high-dimensional images, spectra, and functional readouts, enabling predictive modeling and significantly enhancing analysis throughput. However, challenges remain, including dependence on large, high-quality datasets, the “black-box” nature of complex models, and a lack of standardization. Future efforts will likely focus on developing explainable AI, creating closed-loop intelligent OoCs for adaptive experimentation, and integrating multi-omics data. By transitioning from a passive analytical tool to an active component in experimental design, AI holds the potential to vastly accelerate the application of OoCs in drug development and personalized medicine.

## 3. Offline Detection Technologies in OoCs

Offline detection plays a critical role in OoCs characterization by enabling systematic analysis of key biomolecules within cellular, tissue, and microenvironmental compartments. This approach typically involves endpoint sample collection, providing highly sensitive and specific quantitative or qualitative data to validate biological functions, establish disease models, and support drug screening. Based on analytical targets and resolution, offline detection can be categorized into molecule-specific analysis and omics-based approaches. The integration of these methods ensures comprehensive biological profiling of OoCs, delivering robust molecular insights for drug development, toxicological evaluation, and mechanistic studies of disease pathways.

### 3.1 Molecule-specific detection technologies in OoCs

Molecule-specific detection technologies serve as a core methodological framework for functional validation and dynamic profiling in OoCs. These approaches systematically quantify or semi-quantify gene expression, protein interactions, and metabolite dynamics across cellular, tissue, and microenvironmental compartments, delivering essential data to advance disease modeling and drug discovery. The framework integrates multidimensional analytical strategies: foundational molecular biology techniques enable targeted biomarker validation; high-throughput platforms facilitate parallel multi-parameter analysis; spatial localization methods combined with microscopic imaging resolve molecular distribution patterns; and instrumental analytical techniques comprehensively monitor metabolomic fluctuations. Coordinated application of these methodologies achieves multi-scale functional characterization, bridging genetic regulation to metabolic network dynamics.

Western blot (WB), quantitative PCR (qPCR), and Enzyme-linked immunosorbent assay (ELISA) are key techniques for characterizing molecular biomarkers in OoCs. WB allows precise detection of target proteins, aiding in cell type identification and signaling pathway analysis, particularly in monitoring protein expression changes induced by drug treatments or toxins 142-144. qPCR, using fluorescence signal quantification, offers sensitive gene expression detection, useful for evaluating barrier integrity in BBB-on-a-chip and osteoblast maturation in 3D bone-on-a-chip 145, 146. ELISA, which quantitatively analyzes secreted biomarkers, complements both WB and qPCR. It quantitatively analyzes secreted biomarkers, with applications including urea metabolism assessment in liver-on-a-chip 147, 148, detection of toxicity markers (KIM-1, IL-6) in renal hypoxic reperfusion injury-on-a-chip 149, and dynamic monitoring of glucose-responsive insulin secretion in Organ-on-vascularNet 150. These complementary methodologies establish a multi-level validation framework, supporting the functional characterization and mechanistic analysis of OoCs.

Luminex technology offers the ability to simultaneously detect multiple indicators, making it valuable for dynamic profiling of inflammatory mediators and cytokines 151. It has been used to detect GM-CSF and interleukins (IL-6, IL-8, and IL-10) in the supernatants of vagina-cervix-decidua-OoCs and feto-maternal-interface-OoCs, helping investigate the effects of infection and inflammation 152. In fetal neuroinflammation models, it enables real-time monitoring of IL-8 and IL-1β gradients 153. While it combines high sensitivity with high throughput, its high cost and the need for rigorous standardization remain challenges. Flow cytometry (FACS), using multi-color fluorescence labeling, allows single-cell phenotypic analysis. It has been applied in lung-on-a-chip to assess nanoparticle uptake 154, in lymphoid-on-a-chip for B/T cell activation profiling 155, and in bone marrow-on-a-chip to track hematopoietic stem cell differentiation 156. Large-particle flow cytometry extends its use to viability assessments in liver microtissues and drug toxicity screening 157. Together, Luminex and FACS provide a multidimensional framework for analyzing soluble factors and cellular behaviors in OoCs.

Immunohistochemistry (IHC) and immunofluorescence (IF) are essential techniques for spatial characterization in OoCs. IHC provides stable colorimetric signals for qualitative and semi-quantitative detection of protein distribution at the tissue level, such as analyzing spatial interactions in breast cancer bone-tropic metastasis models and studying neurodevelopmental disorders in BBB-on-a-chip 158, 159. IF, on the other hand, enables subcellular-resolution, multi-target dynamic tracking through fluorescence labeling, as seen in 3D placenta-on-a-chip and liver-gut-on-a-chip models to evaluate cell interactions and metabolism 160, 161. While IHC offers low cost and stable signals, IF excels in sensitivity and multiplexing capability. Together, these methods provide a comprehensive approach for analyzing target protein expression, supporting disease modeling and drug screening in OoCs applications.

In the characterization of OoCs, various instrumental analysis techniques play a crucial role. Attenuated total reflectance fourier-transform infrared spectroscopy (ATR-FTIR) is used to analyze surface functional group changes following chemical modifications. For example, in studying the interaction between PDMS and type I collagen 162, ATR-FTIR effectively reveals newly formed amine and carboxyl functional groups along with their characteristic absorption peaks. This technique provides direct insights into surface chemical structures, aiding in confirming protein immobilization and other chemical modifications. However, its limited penetration depth and sample preparation requirements constrain its broader application. GFAAS enables the precise quantification of metal ions within OoCs, such as nickel ion concentrations in circulating culture media 163, which is critical for assessing system toxicity and immune cell activation. Graphite furnace atomic absorption spectrophotometry (GFAAS) is highly sensitive for metal ion detection but poses challenges due to complex sample preparation and its restriction to specific metal elements. High-performance liquid chromatography with ultraviolet detection (HPLC-UV) allows for quantitative analysis of drug concentration variations in OoCs, facilitating pharmacokinetic studies and tracking drug metabolism in simulated environments 164. However, this method may have limited accuracy in mimicking human physiological processes. Headspace solid-phase microextraction gas chromatography mass spectrometry (HS-SPME-GC-MS) detects volatile organic compounds (VOCs) within OoCs, which can serve as potential tumor biomarkers, reflecting metabolic changes in tumor cells 165. Yet, its application is challenged by stringent sample collection and analysis conditions, as well as the complexity of VOC identification. In summary, these analytical techniques offer unique advantages in OoCs, and characterization, yet each has its own limitations. Selecting and optimizing the appropriate technique based on research objectives and sample characteristics is crucial for enhancing experimental accuracy and reliability.

Molecular-specific detection technologies provide multi-level analyses from genes to metabolites, significantly improving the reliability of OoCs and advancing their applications in precision medicine and drug development. Future research should focus on developing highly compatible, cost-effective detection strategies that accommodate the complex microenvironments and real-time monitoring demands of OoCs.

### 3.2 Omics technologies in OoCs

#### 3.2.1 Application of multi-omics in OoCs

Multi-omics technologies encompass genomics, transcriptomics, proteomics, and metabolomics. They provide systematic quantification of molecules across different biological layers, including RNA, proteins, and metabolites, to unbiasedly reveal complex biological processes. In OoCs' research, integrated multi-omics analysis has become the gold standard for deeply characterizing model fidelity, deciphering functional mechanisms, and evaluating perturbation responses.

Transcriptomics effectively verifies model authenticity in OoCs. In liver-on-a-chip, it confirms correct cell type differentiation by detecting hepatocyte-specific genes, including drug-metabolizing enzymes such as CYP3A4 and transporters like SLCO1B3 166. Similarly, Hart et al. applied transcriptomics in a glomerulus-on-a-chip to precisely reveal molecular interactions between endothelial cells and podocytes. These findings validate the biofidelity of multicellular systems 167. Single-cell transcriptomics further supports the high similarity of intestinal-on-a-chip to human tissues in mimicking intestinal function and pathology 168. For mechanistic insights, proteomics and metabolomics exhibit strong capabilities: analysis of culture medium can directly confirm key liver functions such as drug metabolism and plasma protein secretion. For instance, integrated transcriptomics and metabolomics can elucidate the role of microbial metabolites in mediating host-pathogen interactions 169, while combining proteomics and phosphor-proteomics reveals how food emulsifiers disrupt the intestinal barrier via activation of the PI3K-AKT/MAPK signaling pathways 170. In assessing perturbation responses, multi-omics provides a systemic perspective—transcriptomics accurately identifies drug-induced alterations in lipid metabolism and steatosis in liver-on-a-chip 171 as well as pollutant-triggered inflammatory gene expression changes 172; metabolomics sensitively captures toxicity-related metabolic biomarkers after drug treatment 173, 174 and PM2.5-induced disruptions in cholesterol and bile acid metabolism 160; while integrated metabolomics, proteomics, and transcriptomics comprehensively decode immune-metabolic pathway reprogramming in COVID-19 infection [175]or reveal key mechanisms such as cell proliferation, inflammatory responses, and anti-fibrotic signaling during liver regeneration 176. Moreover, multi-omics technologies enable exploration of multi-organ interactions: proteomics in a neurovascular unit-on-a-chip uncovers the importance of metabolic coupling between endothelial cells and neurons in maintaining brain function 177, and linking pancreatic and liver-on-a-chip combined with transcriptomic and proteomic analysis successfully models diabetes-related metabolic disorders 178.

#### 3.2.2 Integrating multi-omics to address low-volume challenges in OoCs

The microfluidic environment of OoCs typically features microliter-scale culture volumes, yielding limited cell numbers. Consequently, the effluents or cell lysates available for analysis are minute in volume, with total protein content often at the nanogram level. This creates a significant gap compared to the microliter-scale samples and microgram-level protein inputs typically required by conventional omics technologies. The challenge is most acute in proteomics, where the inability to amplify proteins, unlike nucleic acids, presents a major bottleneck 179.

In proteomics, the inability to amplify proteins or peptides necessitates meticulous optimization across the entire workflow, including collection, enrichment, processing, and acquisition, to maximize the recovery and detection of informative molecules. During sample collection and preprocessing, the priority is to minimize adsorptive losses and evaporation. In practice, the use of low-binding microtubes and immediate sample freezing is recommended. Furthermore, pooling samples from multiple chip units or extending collection periods effectively increases the total protein amount. For instance, in an open microfluidic vessel-on-a-chip, pooling cells from six arrayed units or collecting 24-hour secretions accumulated sufficient protein for subsequent analysis 180. Similarly, in an adipose micro-tissue-on-a-chip, non-destructive retrieval and pooling of 12 micro-tissues elevated peptide concentrations to a range detectable by the Q-Exactive HF-X mass spectrometer 181. Techniques like vacuum centrifugation concentrate microliter-scale liquids, while optimized chip designs (e.g., open reservoirs and retrievable hydrogels) help reduce sample retention 182.

For enrichment and processing, proteomics relies on highly efficient concentration and purification schemes. The SP3 magnetic bead method is widely adopted for its efficient capture and purification of trace proteins, demonstrating excellent performance in both spinal cord and cardiovascular OoCs 180, 183. Micro-solid-phase extraction tips, such as stop and go extraction tips (StageTips), are routinely used for peptide desalting and cleanup. To minimize transfer losses, on-chip digestion strategies can be employed, exemplified by the micro suspension trapping column approach used in a human cervix-on-a-chip 184. Standardized kits facilitate uniform digestion and purification from nanogram-level inputs, ensuring reproducible peptide generation. EvoTip, a specialized StageTip, features low-adsorption PTFE material and an automation-friendly design 185. For future development, it could be directly integrated into the open reservoir module at the outlet of OoCs. This integration would enable *in situ* capture of secreted proteins or lysates, thereby minimizing sample loss from transfer steps. Furthermore, for OoCs incorporating 3D hydrogel scaffolds, an adapted “*in situ* digestion-capture” workflow could be developed. This approach would effectively reduce matrix-induced interference and enhance protein identification.

Finally, for data acquisition, state-of-the-art high-resolution mass spectrometry platforms (e.g., Orbitrap Q Exactive HF/X series) are coupled with nano-reversed-phase liquid chromatography (nano-RSLC). These systems are operated in highly sensitive modes, such as data-dependent acquisition (DDA) or data-independent acquisition (DIA), with optimized settings for low-abundance peptide detection, enabling the identification of hundreds to thousands of proteins 186. Wang et al. employed nano-LC-MS/MS for proteomic profiling of the minimal samples derived from OoCs. This platform, with its high resolution and DDA, achieved extensive proteome coverage from limited material. The analysis successfully identified key differentially expressed proteins, including MFAP2 and LTBP-1. These findings validated the molecular authenticity of the Angiotensin II-induced cardiomyopathy model. Furthermore, the study delineated, for the first time on OoCs, the multi-target anti-fibrotic mechanisms of relaxin 187. Looking forward, the next frontier for OoCs proteomics lies in integrating spatial and single-cell resolutions. Spatial proteomics techniques, while still in early stages of adaptation to OoCs, could map protein expression and post-translational modifications to specific regions within a miniature tissue construct, such as distinguishing the proteome of a vascular channel from an adjacent parenchymal chamber 188. Similarly, advances in single-cell proteomics by mass spectrometry (SCoPE-MS) and its derivatives promise to deconstruct the cellular heterogeneity within OoCs, identifying rare cell types or stochastic cellular responses to perturbations that are masked in bulk analyses 189.

In contrast, genomics and transcriptomics benefit from powerful amplification techniques. These methods can generate sufficient sequencing material from even a few cells, effectively circumventing the low-input challenge inherent to OoCs 190. Metabolomics also faces low-concentration challenges, but sample pretreatment is often simpler (e.g., protein precipitation), and modern MS-based detection offers sufficient sensitivity for microliter-scale samples. In summary, overcoming the low-input bottleneck in OoCs' multi-omics, particularly for proteomics, is being achieved through integrated miniaturized sample handling, efficient enrichment strategies, and advanced detection platforms.

In the research of OoCs, material innovations for molecular-specific detection and omics analysis are breaking through along multi-dimensional paths. Through the development of implantable micro-capture devices, anti-fouling microfluidic interface chips, and intelligent stimulus-responsive enrichment media, combined with biomimetic recognition materials to solve the specificity bottleneck of molecular detection, and by leveraging nano-confined separation materials to achieve omics-scale panoramic analysis of micro-samples, an exclusive offline detection material toolkit for OoCs is ultimately constructed. These advancements will drive offline detection to evolve from endpoint analysis toward quasi-dynamic monitoring, forming a synergistic closed loop with fluorescent probe/sensor technologies to provide OoCs with cross-scale verification capabilities from molecular events to organ functions.

## 4. Conclusion

As an innovative *in vitro* simulation platform, OoCs characterization has matured into two complementary paradigms: offline and online detection systems. Together, these approaches propel advances in disease modeling and drug development. Offline detection focuses on molecular-specific biomarker analysis and multi-omics profiling, employing high-resolution analytical instruments like mass spectrometry and sequencing to deeply interrogate biological signatures and unravel systems-level disease mechanisms. Integrated metabolomic and proteomic strategies exemplify this approach, constructing molecular fingerprints while enabling precision medicine applications. However, these techniques face inherent limitations, including complex sample processing, operational delays, and environmental perturbations, making real-time monitoring of dynamic biological processes challenging. Conversely, online detection integrates biosensing and fluorescent probe technologies to enable continuous, real-time monitoring of OoCs. Biosensors offer high sensitivity and specificity for tracking biomarker fluctuations, facilitating rapid assessment of drug responses and biological functions, while fluorescent probes provide high-resolution, non-invasive visualization of microenvironmental dynamics and cellular behaviors. The integration of AI further augments this framework through its ability to leverage ML or DL algorithms for complex data analysis. This reveals hidden patterns within intricate datasets, generating novel insights for OoCs optimization and experimental design.

While the integration of multiple real-time monitoring probes, as reviewed herein, undoubtedly enriches the data output from OoCs, it is crucial to acknowledge the inherent trade-offs and limitations. Firstly, the physical incorporation of probes and sensors can disrupt delicate 3D tissue architectures, while the use of non-biological materials may compromise native cell-cell and cell-matrix interactions fundamental to organ function 23, 191, 192. Consequently, a critical balance must be struck between data density and biological fidelity. Secondly, the platform-specific nature of these technologies presents a significant challenge. Many advanced analytical techniques are uniquely tailored to particular chip architectures. For instance, in the BBB-on-a-chip developed by Sujey et al., the micro-TEER measurements are highly dependent on the precise placement and design of the electrodes, a feature that varies substantially across different platforms 110. Similarly, the efficacy of optical imaging modalities can be limited by a chip's material opacity and internal geometry 193. Therefore, a probe or assay optimized for one platform is seldom directly transferable to another due to profound differences in materials, dimensions, and fluidic principles. Ultimately, the metrics generated from any OoCs are contextualized by these design choices and technological constraints. Acknowledging these challenges is a necessary step for the field's maturation.

Looking forward, the consensus is that OoCs' characterization is evolving towards sophisticated, non-invasive, real-time monitoring, yet its widespread implementation faces systemic challenges. Key priorities include developing high-sensitivity probes and nanoscale sensors for the* in situ* tracking of metabolites and ion concentrations. Simultaneously, multi-parameter sensing platforms must overcome the limitations of single-parameter detection to provide a holistic functional analysis. For real-time monitoring, integrating miniaturized biosensors with intelligent microfluidics will enhance spatiotemporal resolution. AI-driven microelectrode arrays, for example, could decode neuro-electrophysiological networks, while dynamic fluorescence imaging combined with ML feedback systems could shift OoCs from static simulation towards adaptive control. Nevertheless, two major obstacles persist: optimizing the long-term stability and biocompatibility of probes/sensors, and developing interpretable AI models for the real-time fusion and analysis of multi-source heterogeneous data. Most crucially, the absence of standardized frameworks severely impedes technological adoption. An urgent priority is establishing cross-laboratory standards for hardware interfaces, sensor calibration, and spatiotemporal metadata architectures, alongside promoting data sharing and algorithm validation through open-source platforms. Ultimately, all technological progress must be anchored in clinical relevance. This goal requires demonstrating strong correlations between the dynamic data from OoCs and human pathological mechanisms, potentially through multi-center studies, and quantifying how accurately OoCs can predict drug toxicity. It is believed that through ongoing technological refinement, OoCs will mature into robust and dependable tools for scientific research, facilitating advances in disease understanding and therapeutic development, all directed toward improving human health.

## Figures and Tables

**Figure 5:**
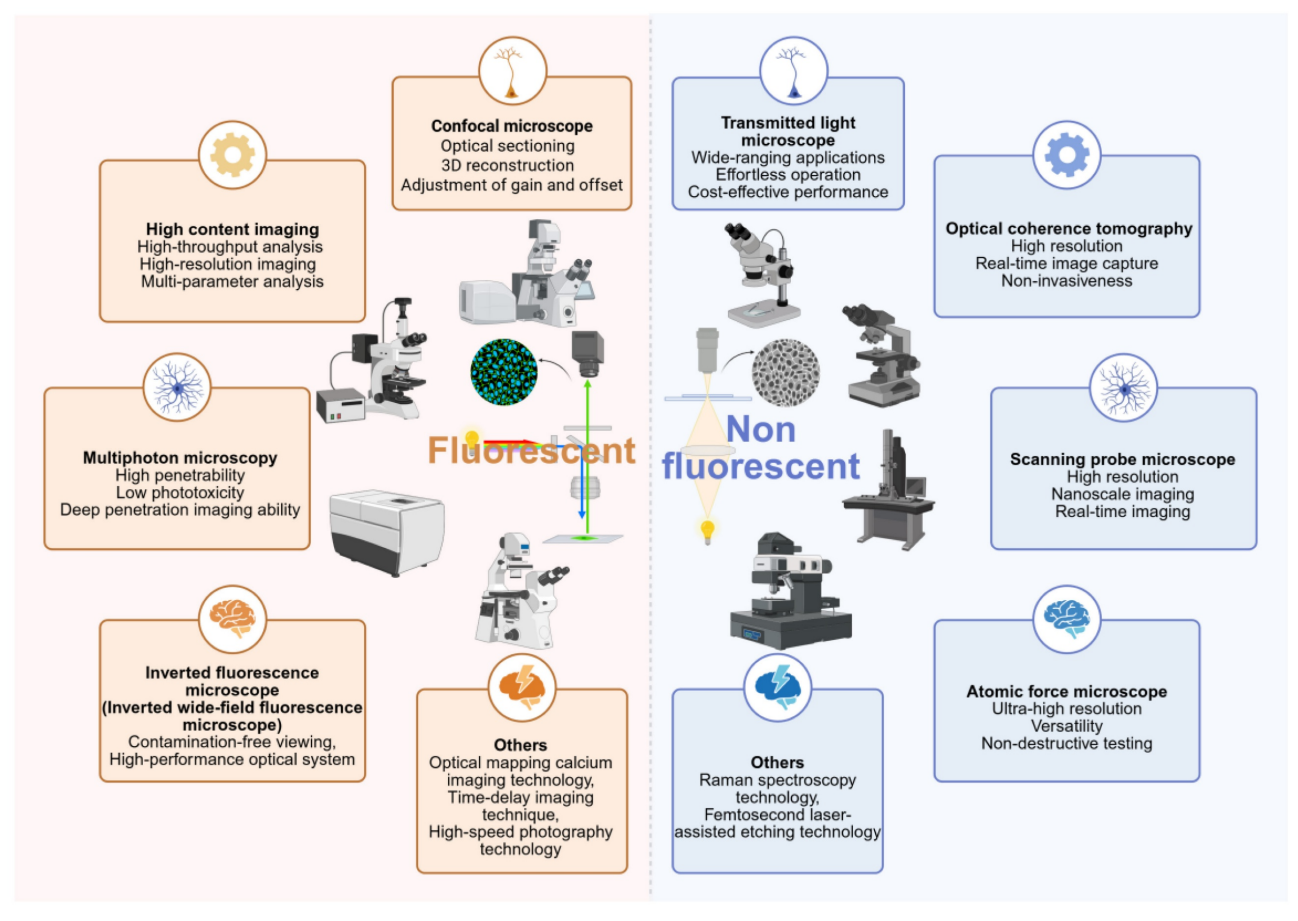
**Recent morphological analysis used in visual recognition of OoCs.** Advancements in microscopy have greatly facilitated research on cell-cell interactions, cellular behaviors, and dynamic changes within the microenvironment. (Created with BioRender.com).

**Figure 6:**
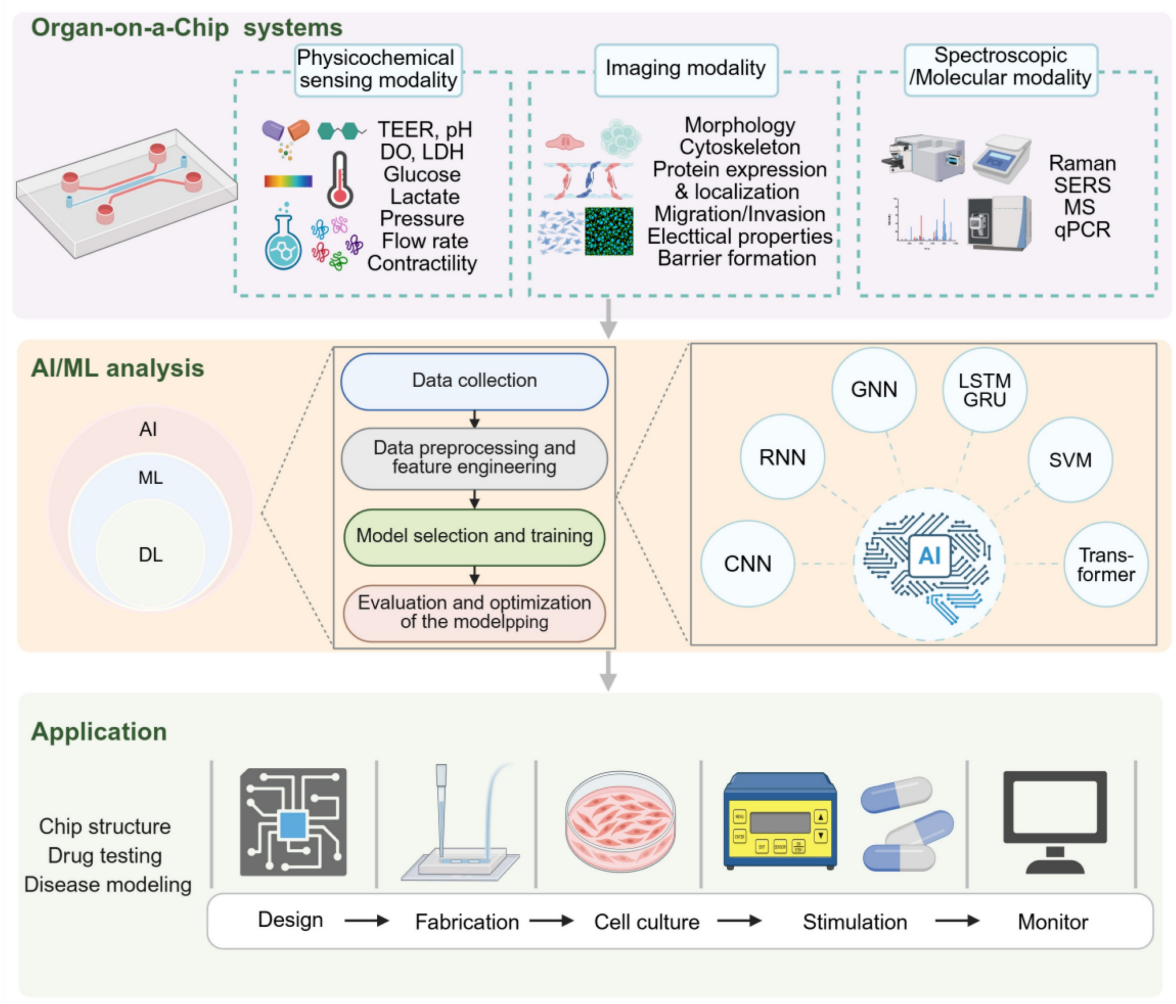
** Workflow for integrating AI with OoCs data analysis.** OoCs generate multimodal datasets, including images (e.g., cellular morphology), non-imaging sensor data (e.g., spectra), and multi-parametric functional readouts (e.g., cell viability). These data undergo preprocessing and feature extraction. Subsequently, they are fed into various AI/ML models to perform specific tasks, such as classification, prediction, and segmentation. Ultimately, the model outputs are translated into biological insights, which help validate hypotheses or guide further experimental design, establishing an iterative, closed-loop optimization process. (Created with BioRender.com).

**Figure 7:**
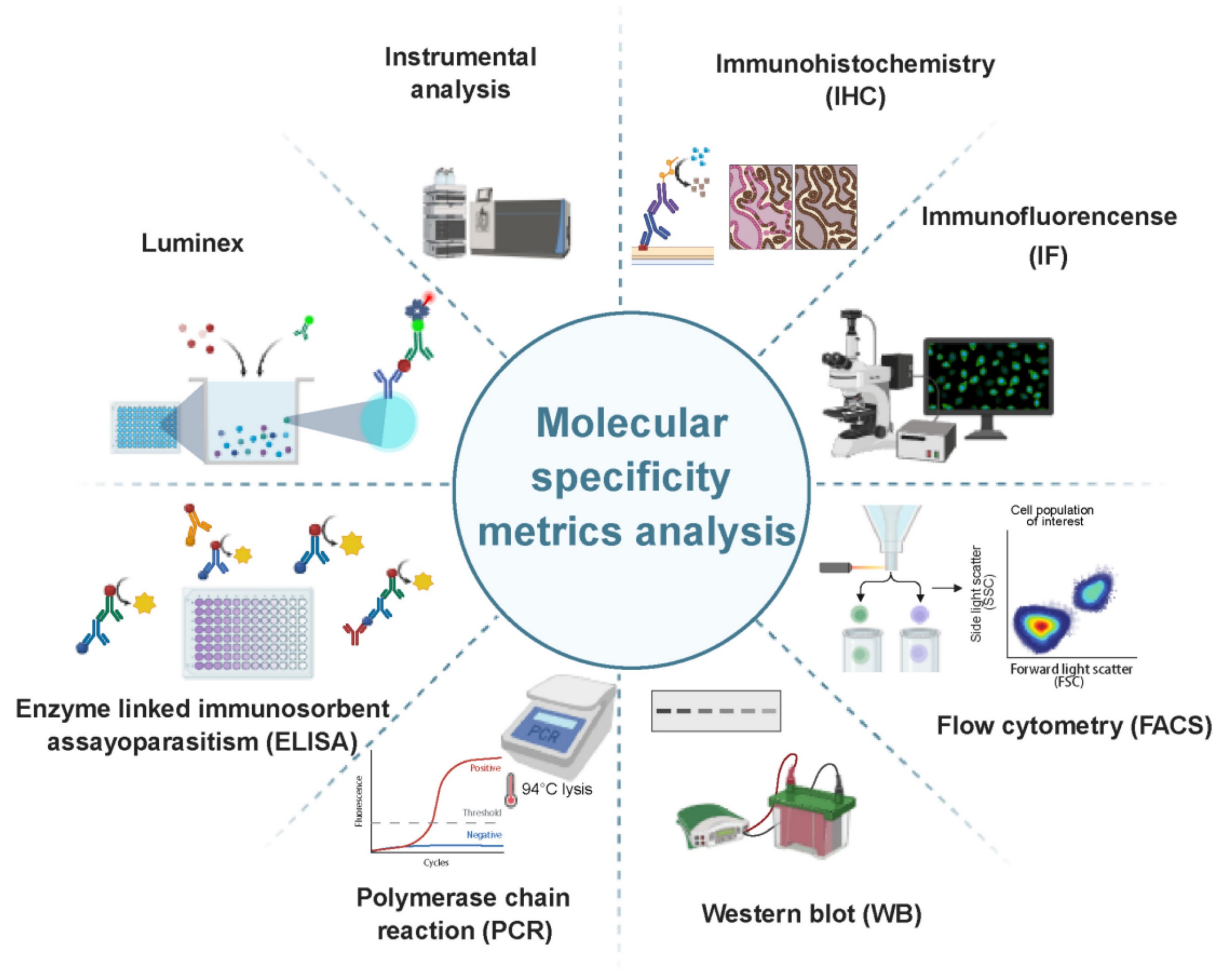
**The applications of molecule-specific technologies in OoCs.** Quantitative or qualitative detection of expression levels and dynamic changes of specific genes, proteins and metabolites to reveal biological functions and response characteristics in OoCs. (Created with BioRender.com).

**Figure 8:**
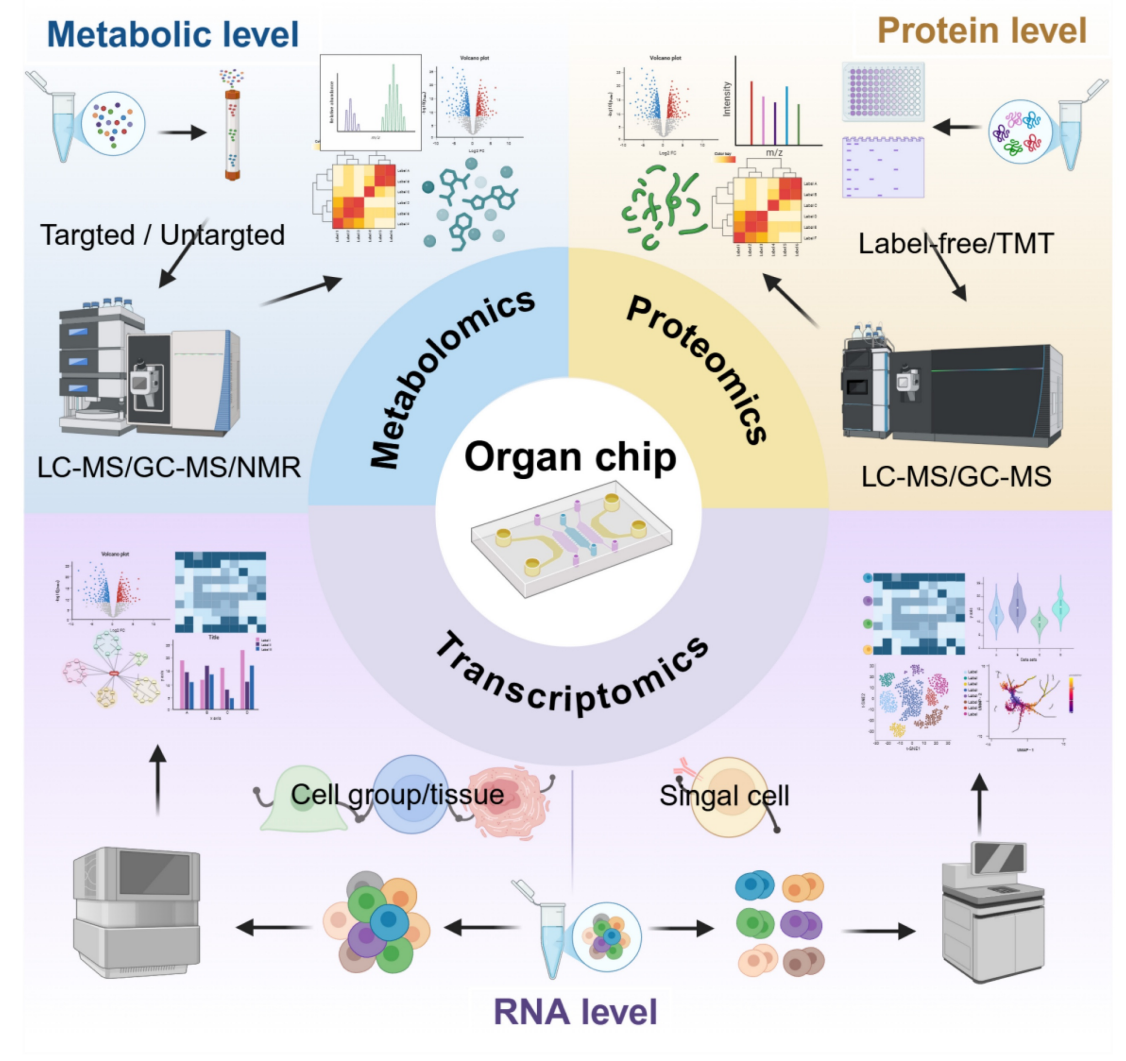
**The applications of omics analysis in OoCs.** Metabolomics employs LC-MS, GC-MS, and NMR to holistically map metabolite dynamics and pathway regulation. Proteomics systematically deciphers protein expression profiles, post-translational modifications, and interaction networks. RNA-Seq-based transcriptomics enables genome-wide transcriptomic profiling, precisely identifying cell subpopulations and functional states. scRNA-seq transcends the resolution limits of conventional assays, deeply resolving cellular heterogeneity, gene expression dynamics, and disease-related molecular mechanisms within OoCs microenvironment. (Created with BioRender.com).

**Table 1:** The summary of biosensors applied in OoCs.

Sensor Classification	Organs	Cell types	Material & Technique	Target Parameter	Ref
Optical	Live	HepG2	Pt (OEP)	Oxygen	27
Optical	Lung	A594, hASC, HUVEC	Pt-TPTBPF	Oxygen	28
Optical	Heart	hiPSC, phDF	Pt-TPTBPF	Oxygen	29
Optical	Intestine	Caco-2	Pt-TFPP	Oxygen	30
Optical	Intestine	Intestine organoid, HIMEC	Oxygen-sensing nanoparticles	Oxygen	31
Optical	Intestine	Intestine organoid, HIMEC, Caco-2	Oxygen sensitive and optical isolating particles	Oxygen	32
Optical	Lung	16HBE	Silicon nitride photonic ring resonators	CRP, IL-1β, IL-6, IL-8	34
Electrochemical	Endothelial	PAECs	Au/Cr electrodes	TEER	39
Electrochemical	Vascular	HUVEC	Au/Cr electrodes	TEER	41
Electrochemical	Endothelial	HUVEC	Pt electrode	Imaging of Endothelial Permeability	42
Electrochemical	Retina	HREC, SH-SY5Y, ARPE-19	Ti/Pt/Ti electrodes	TEER	43
Electrochemical	Gut	Caco-2	Gold-plated electrodes, stainless steel wires	TEER	44
Electrochemical	Placental	Bewo	Impedance electrodes	TEER	46
Electrochemical	Lung	16HBE14	Cu covered with Ni electrode, Au electrode	TEER	47
Electrochemical	Gut	Caco-2, RPTEC	Electrode board	TEER	48
Electrochemical	Heart	hiPSC	Au/hydrogel pillar electrodes	Cardiac tissues electrophysiology	50
Electrochemical	Heart	hiPSC-CM, Lonza	µECG	Cardiac tissues electrophysiology	51
Electrochemical	Heart	HL-1	Au/Pt nanopillar	Cardiac tissues electrophysiology	52
Electrochemical	Heart	hiPSC	Pt (PEDOT) electrode, Au electrode	Electrophysiological mapping	53
Electrochemical	Spinal	Human stem cell-derived sensory-spinal cord organoids, human dorsal spinal cord interneurons, sensory neurons	Cytoview MEA 24	Human nociceptive circuitry	55
Electrochemical	Tongue	Taste organoid	MEA 2100 system	Extracellular potential signals	56
Electrochemical	Islets	Islets	MEA, m-MEA	Islet ion fluxes	57
Electrochemical	Gut	HUVECs, HLECs	TEER electrode, O_2_ sensors, pH sensors	TEER, Oxygen, pH	74
Optical Electrochemical	Liver	HepG2/C3A	Polystyrene microbeads loaded with ruthenium-based phosphorescence dye (CPOx-50-RuP), Platinum electrodes	Oxygen, glucose, lactate	75
Optical	Lung	A549	ptBS particle with Pt-TPTBPF, OHC12 dye/Egyptian blue	Oxygen, pH	76
Electrochemical	Gut	Caco-2	Ag/Cl electrodes, three-electrode electrochemical sensors	TEER, Hg (II)	77
Electrochemical	Heart	HUVECs, hiPSC-CMs	Au electrode, MEA (platinum black)	TEER, Cardiac tissues electrophysiology	78
Optical Electrochemical Physical	Heart-liver	Cardiac/liver organoids	Au electrode, phenol red, [Ru(dpp)3]^2+^ Cl^2-^tris(4,7-diphenyl-1,10-phenanthroline) ruthenium (II) chloride, Type T thermocouple Probe	Albumin, pH, oxygen, temperature	79
Optical Electrochemical Electrophysiological	Neurons	Neuronal cell	TFT	Optical observation, impedance measurement, LFP, AP	80
Electrochemical	Liver-heart-lung	HSC, Kupffer, iPSC CM, TMSCS, HTBS	Au electrode (SAM-SPV), customized TEER electrode	ALB, GSTA, CK, TEER, Isc	81
Electrochemical	Liver	Primary hepatocytes	Au/Ag electrodes	Oxygen	82
